# Virtual tactile feedback technology based on microcurrent stimulation: current status, challenges and future prospects

**DOI:** 10.3389/fnins.2025.1519758

**Published:** 2025-05-09

**Authors:** Qiwei Xiong, Hongbo Yao, Jiyu Wang, Zhenghao Huang, Junhong Luo, Changfu Zhong, Yihuan Lin, Lin Shu

**Affiliations:** ^1^School of Electronic and Information Engineering, South China University of Technology, Guangzhou, China; ^2^School of Future Technology, South China University of Technology, Guangzhou, China

**Keywords:** human-computer interaction, electrotactile feedback, virtual reality, virtual tactiles, audiovisual-tactile integration

## Abstract

With the rapid development of information technology, virtual reality (VR) technology and metaverse, which highlight personalized experience, have become hot spots in the development of information application industry. Visual, auditory, and tactile systems are the most common sensory systems used by human beings to perceive information about the external environment, facilitated by organs such as the eyes, ears, and skin, making it convenient and natural to interact with the outside world. The integration of virtual tactile feedback technology with audiovisual technology can further enhance the richness of interaction and achieve better immersion experience. Among the many tactile feedback technologies, electrical stimulation tactile feedback stands out due to its performance advantages such as device portability, high refresh frequency and precise control. However, electrical stimulation technology lacks a mature three-dimensional human tissue electrical stimulation conduction model with multipoint stimulation in theoretical research; the variety of virtual tactiles implemented in the research is limited, and there is a gap with real life; and there are fewer audio-visual tactile feedback fusion control models and equipment development problems. Based on these challenges, this paper combs through the latest research progress of microcurrent stimulation-based virtual tactile feedback technology in the field of human-computer interaction. “Microcurrent” here refers to the application of low-intensity electrical currents, specifically under 10 milliamperes, which provide precise and adjustable stimulation for enhancing tactile experiences in virtual and augmented reality applications. This summary outlining the technical characteristics and current research status of this research direction. Finally, the current problems and future development trends in this field are discussed in depth, and how to improve them in order to develop a broader application space is analyzed. By clarifying the potential value of tactile feedback in human-computer interaction, it is hoped to promote the future development of electrically stimulated tactile feedback technology in human-computer interaction, and to help develop a more natural, realistic, efficient and immersive human-computer interaction experience. This study uniquely integrates a systematic analysis of electrotactile perception mechanisms with emerging microcurrent stimulation technologies, providing practical guidelines and a novel reference framework for future research in virtual haptics.

## 1 Introduction

With the rapid development of information technology, modern lifestyles are becoming fast-paced and human-centered. This fast pace has fostered virtual-reality fusion technologies, while the human-centered trend has increased demand for personalized information applications. As a result, virtual reality (VR) technology and the metaverse have become focal points in the information application industry due to their outstanding personalized experiences. Human-computer interaction (HCI) technology has gained significant attention for enabling virtual-reality integration and meeting personalized user needs.

HCI involves the communication and exchange of information between users and machines, including computers, mobile devices, and software systems, to facilitate task completion. The evolution of HCI has progressed through distinct stages, from manual operations and command-line interfaces to graphical user interfaces, web-based interfaces, and the current phase characterized by multi-channel and intelligent interaction. In the contemporary era, HCI is advancing towards multimodality and intelligence, enabling more natural and efficient interactions through the integration of visual, auditory, and tactile modalities.

In multimodal human-computer interaction, virtual tactile technology allows users to experience interaction targets in real time by simulating tactile features such as texture, vibration, and temperature. This enhances operational efficiency (Svensson et al., 2017; Dangxiao et al., 2019; Yao et al., 2022). In recent years, the field of virtual tactile feedback in HCI has made significant progress thanks to the efforts of many research workers. For example, through the integration of wearable gloves and other devices, tactile feedback can provide a better immersion experience (Zhou et al., 2022). In clinical medical training, critical equipment operation training and other professional fields, tactile feedback can strengthen the operator’s “hand-eye coordination” ability (Patel et al., 2022). In general-purpose scenarios like smartphones and computers, HCI has evolved rapidly. It has shifted from keyboards and mice to multi-touch and from voice interaction to multimodal systems. However, challenges remain, such as the high complexity of input interactions and the limited feedback confined to visual and auditory senses. These limitations reduce immersion and telepresence. Introducing tactile feedback is essential to meet the growing demand for personalized and immersive experiences. Recent advancements such as high-resolution electrotactile arrays (Lin et al., 2022) and adaptive tactile rendering based on physiological feedback (Yao et al., 2022) have significantly improved the realism and adaptability of virtual tactile systems (Yao et al., 2022; Lin et al., 2022).

In the field of HCI, traditional tactile feedback devices often rely on piezoelectricity, airbags, or similar modalities to achieve tactile feedback. However, these devices are typically large, bulky, inconvenient to operate, and complex to wear (Pylatiuk et al., 2006; Varga, 2018; Perret et al., 2017). In contrast, virtual tactile rendering technology based on microcurrent stimulation offers several advantages, including simplicity of implementation, lightweight and flexible design, ease of wear, and precise control of stimulation parameters (Lin et al., 2022; Wang et al., 2021; Strbac et al., 2016). Additionally, this technology effectively stimulates a broader range of skin receptors, enabling the generation of diverse tactile sensations. As a result, it holds significant potential in applications such as virtual reality, teleoperation, medical care, entertainment, and education (Al-Ayyad et al., 2023). In this paper, we will describe the basic operating procedures and applications of the microcurrent stimulation technique, outline the application cases in this field in recent years, and discuss the areas that need to be improved in the process of research and use. Through this work, this paper aims to introduce this emerging technology, promote its integration and application in related fields, and provide new perspectives and ideas for various researchers in research and innovation in this field.

## 2 Methodology

**Figure 1:**
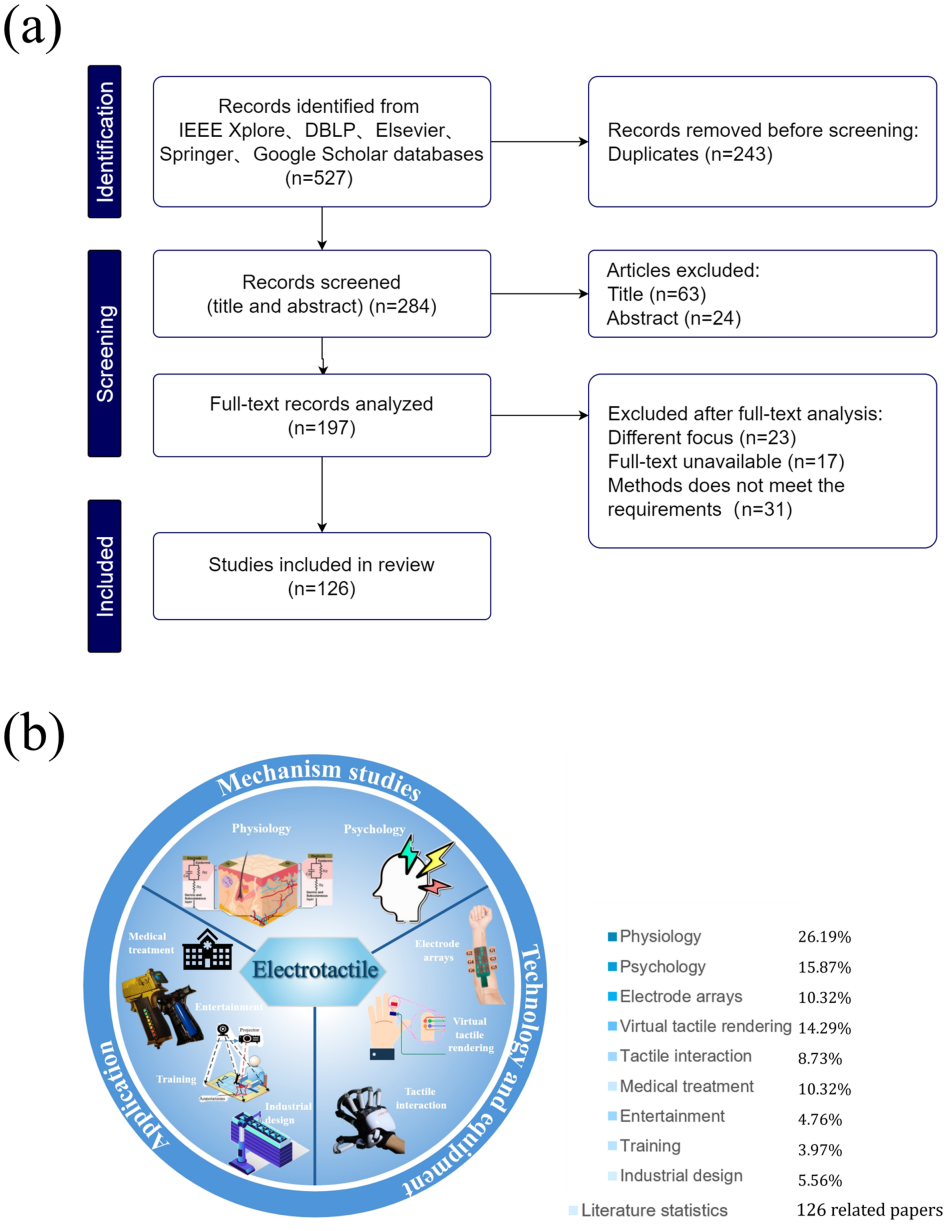
Literature statistics. **(a)** Study selection using PRISMA flowchart. **(b)** statistical distribution of topics in electrotactile research literature.

This review is conducted as a systematic review and strictly adhered to the Preferred Reporting Items for Systematic Reviews and Meta-Analyses (PRISMA) extension guidelines to ensure methodological rigor and transparency, as illustrated in Figure 1a. As shown in Figure 1b, a systematic search was conducted across five databases to retrieve studies related to the field of electrotactile research. After screening based on predefined inclusion and exclusion criteria, 126 out of an initial 527 articles were ultimately selected for in-depth analysis and synthesis. The screening process was carried out in two stages. In the first stage, titles and abstracts were independently screened to exclude irrelevant or duplicate records. In the second stage, full texts were reviewed to determine eligibility. Disagreements during either stage were resolved through team discussion, and a third team member was consulted when necessary to reach consensus. To ensure quality, only studies with demonstrable methodological soundness and clear relevance to the research objectives were included, as further detailed in section 2.2.

### 2.1 Search strategy

A total of 527 articles were collected from databases such as IEEE Xplore, dblp, Elsevier, Springer, and Google Scholar. Terms related to electrotactile features or conditions were chosen and combined with specific electrotactile terms to filter relevant articles. Electrotactile terms included: electrode arrays (or surface electrode layouts, or electrode distributions); electrotactile rendering (or electrotactile images, or electrotactile simulation); electrotactile interaction (or electrotactile feedback); virtual tactile rendering; and tactile psychology (or tactile physiology). In addition to database searches, reference lists of selected papers were checked to identify studies potentially missed in the initial search.

### 2.2 Inclusion and exclusion criteria

Articles were considered from three perspectives: studies on the mechanisms of electrotactile feedback, technology development, and practical applications. In the first screening stage, duplicates and irrelevant articles were excluded. Full texts were reviewed after assessing titles and abstracts, excluding papers that were not focused on electrotactile research, to ensure the inclusion of studies analyzing the physiological and psychological effects of electrotactile interaction in human-machine interfaces. In the second stage, reliable studies were selected by prioritizing publications from peer-reviewed journals, with a focus on author credibility, methodological rigor, and result consistency. These criteria were applied to ensure the inclusion of reliable, high-quality sources. However, given the specific scope of the selected literature, primarily focusing on studies published in peer-reviewed journals and prominent conferences, there might be inherent limitations regarding the representativeness of the selected sample. Particularly, studies involving cutting-edge experimental setups or those published in less accessible venues could have been underrepresented, potentially affecting the generalizability of this review.

## 3 Virtual tactiles research

Research in the field of virtual haptics has been attracting a lot of attention, especially in the research direction closely related to electrically stimulated tactile feedback technology. In this paper, we will explore several key areas of electrostimulation tactile feedback technology, ranging from the mechanisms of electrotactile perception to the design and application of related devices. Firstly, we will focus on the perception and physiological mechanisms of electrotactile sensation in the human body, a fundamental knowledge that is essential for understanding how electrotactile feedback works and its effects. Subsequently, we will analyze key factors such as the perceived quality, intensity and comfort of electrically stimulated haptics. Further, this paper will review the current state of research on virtual haptic messaging and resolution. Additionally, it will explore recent advancements in haptic interaction devices (Lin et al., 2022; Warren et al., 2008; Boldt et al., 2014). Comprehensive research in these areas provides important guidance for the development of more natural and efficient human-computer interaction technologies. Next, we will explore the current research status of each of these key areas in depth.

### 3.1 Research on the physiological and psychological mechanisms of electrotactile sensation

#### 3.1.1 Current research on physiological mechanisms of electrotactile sensation

##### 3.1.1.1 Research on skin and receptors

Skin, one of the largest and most regenerative structures in the human body, performs essential functions such as protection, sweating, and sensing external stimuli like heat, cold, and pressure. As the body’s primary sensory organ, the skin contains various types of receptors that detect both internal states and external stimuli. These receptors convert sensory signals into nerve impulses, which are transmitted via the nervous system to the brain’s cortical areas, forming corresponding perceptions.

The human body’s sense of touch is generated through the mechanoreceptors within the skin in response to external stimuli. The skin and subcutaneous soft tissues contain four main types of mechanoreceptors, namely: Meissner’s tactile corpuscles, Merkel’s tactile discs, Pacinian annular lamellipodia, and Ruffini’s corpuscles. These receptors are distributed in the tissues of the skin at different depths.

Meissner’s corpuscles, located in the superficial layers of the skin, have a spatial resolution of 3–5 mm. They are highly sensitive to skin deformations (Folgheraiter et al., 2008). The mechanoreceptors distributed at the base of the epidermis are Merkel tactile discs, which are connected in a disc-like fashion, often adjacent to Meissner tactile corpuscles. Merkel tactile discs have a very good perception of the surface properties of the skin, such as thickness, texture, and tensile changes. Although their response frequency is not high, their high spatial resolution of 0.4 to 0.6 mm makes them extremely responsive to dynamic stimuli (Kajimoto et al., 2004). Deeper into the skin tissue, the mechanoreceptors distributed in this location are Pacinian annular lamellipodia, whose ring-type structure is responsive to stimuli at 60–80 Hz and very sensitive to deformation of skin such as vibration and friction of the skin (Folgheraiter et al., 2008). And the mechanoreceptors distributed in the dermis of the skin are Ruffini’s corpuscles, and these receptors respond at frequencies similar to those of Merkel’s tactile discs, with a wide range of sensory areas but low precision. Each mechanoreceptor corresponds to a specific tactile experience, and only when multiple receptors are activated at the same time can the human body obtain rich tactile sensations (Johnson et al., 2000).

Based on the physiological characteristics of human skin receptors, the four skin receptors mentioned above can be grouped into two main categories (Delmas et al., 2011): rapidly adapting receptor (RA or FA) and slowly adapting receptor (SA). Rapidly adapting receptors include Meissner tactile corpuscles and Pacinian annular layer corpuscles, while slowly adapting receptors consist of Merkel tactile discs and Ruffini’s corpuscles (Kajimoto et al., 2004). In addition, each type of receptor can be further subdivided into type I and type II. Specifically, Meissner tactile corpuscles are classified as RA type I, Merkel tactile discs are SA type I, Pacinian annular lamellipodia belong to RA type II, and Ruffini’s corpuscles are SA type II. The main difference between these two major types of receptors is the duration of their response to a stimulus. Fast-adapting receptors adapt to stimuli quickly but briefly, while slow-adapting receptors adapt slowly and persistently.

In the current electrotactile research, since the contribution of Ruffini’s corpuscles to tactile perception is relatively small, researchers have mainly focused on the response of the other three receptors to external stimuli (Butikofer and Lawrence, 1979), which are figuratively referred to as “tactile primary colours” by Kajimoto et al. (2004). This nomenclature implies that there is a similarity between the formation of tactile sensation and the mixing of colours. Just as a variety of colours can be produced by different combinations of the three primary colours of red, green and blue, a wide variety of tactile experiences can be created by stimulating different combinations of these three receptors, thus providing a rich variety of tactile perceptions.

##### 3.1.1.2 Research on neurons

**Figure 2:**
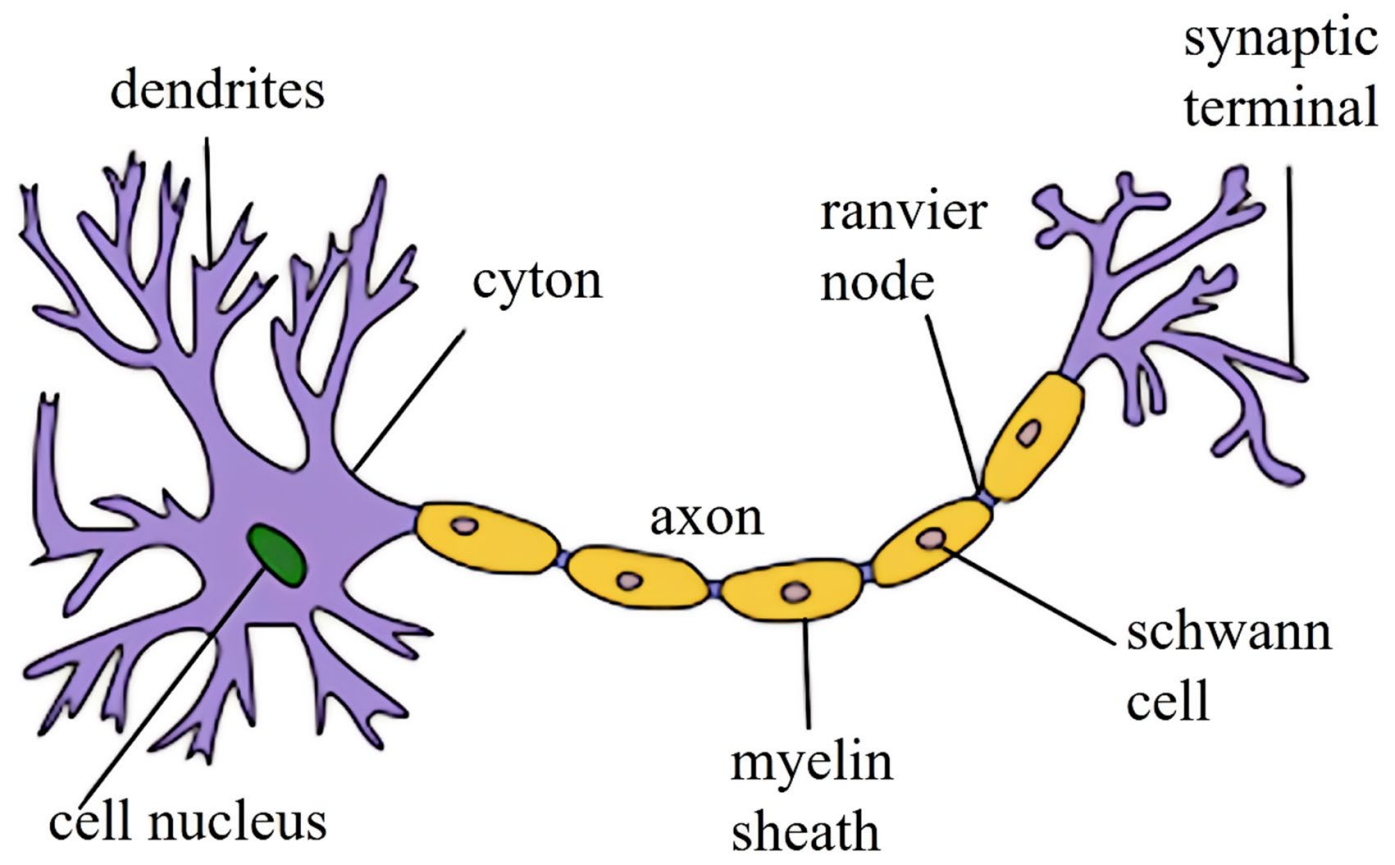
Neuronal structure.

Electrical stimulation activates tactile receptors, generating bioelectrical signals that trigger sensory nerve fibres to transmit information. Additionally, it can directly stimulate sensory neurons, inducing nerve impulses. These signals are further transmitted to the brain’s central nervous system. The human brain contains nearly 100 billion neurons, interconnected through synapses to form complex neural networks. These networks perform essential functions, including information transmission, processing, integration, and computation. The basic structure of neurons, comprising dendrites, axons, and soma, is illustrated in Figure 2.

The relationship between electrotactile physical stimulation, peripheral nerve activity, central nerve activity, and electrotactile perception was first investigated by Kaczmarek et al. (2000) using recording of action potentials from three RA fibres innervating primate fingerpad. The study designed two experiments in which mechanical stimulation was coupled with electrical stimulation. In the first experiment, the current intensity was gradually increased from 0 to the threshold value and beyond, while recording three RAI neuron discharges. The results showed a sudden increase in neuron discharge rates at the threshold value. However, further increases in current intensity did not significantly alter the discharge rate. The second experiment applied electrical stimulation with a 20 μs pulse width and sinusoidal mechanical stimulation at 30 Hz simultaneously. The differences in RA neuronal discharge were recorded before and after the electrical stimulation. The results showed that single mechanical stimulation induced neuronal discharge of about 10 AP/s. When superimposed on electrical stimulation, the same neuronal discharge abruptly changed to 30 AP/s. Experimental results show that electrical stimulation plays an important role in tactile perception, both independently triggering neuronal responses and synergistically with mechanical stimulation to significantly enhance neural activity. Kaczmarek’s study provides key evidence for understanding the physiological mechanisms of electrotactile sensation, demonstrating the potential and benefits of electrical stimulation in the optimisation of tactile feedback devices and the enhancement of user experience.

#### 3.1.2 Research on the modelling of electrotactile bioelectrical signals

Research on the biological model of electrotactile perception provides valuable insights into its underlying mechanisms. These models allow researchers to study how electrical stimulation propagates through the skin and nerves, offering a deeper understanding of the core principles of electrotactile technology. This knowledge is crucial for guiding the design and optimisation of electrotactile systems. Based on the generation mechanism of electrotactile sensation, this section categorises bioelectric signal modelling into three areas: skin impedance modelling, electrode-skin contact modelling, and neuron conduction modelling.

##### 3.1.2.1 Skin impedance modelling

Early research on skin impedance models mainly focused on the measurement of skin impedance, such as Schwan (1968) studied the polarization impedance and measurement of electrodes in biomaterials. Further research revealed that the skin’s resistive properties under electrical stimulation are primarily attributed to hair follicles and sweat glands, while its capacitive properties are associated with lipid bilayers. This dual nature gives skin impedance distinct resistance-capacitance characteristics. Consequently, researchers developed standard electrical equivalent models of the skin by simulating its impedance using resistance-capacitance (RC) circuits. For example, van Boxtel (1977) had already started to use resistive-capacitive parallel networks for the equivalent of skin resistance in his research to more closely reflect the electrical behavior of the skin under the action of a 1–10 mA square wave electrical pulse.

**Figure 3:**
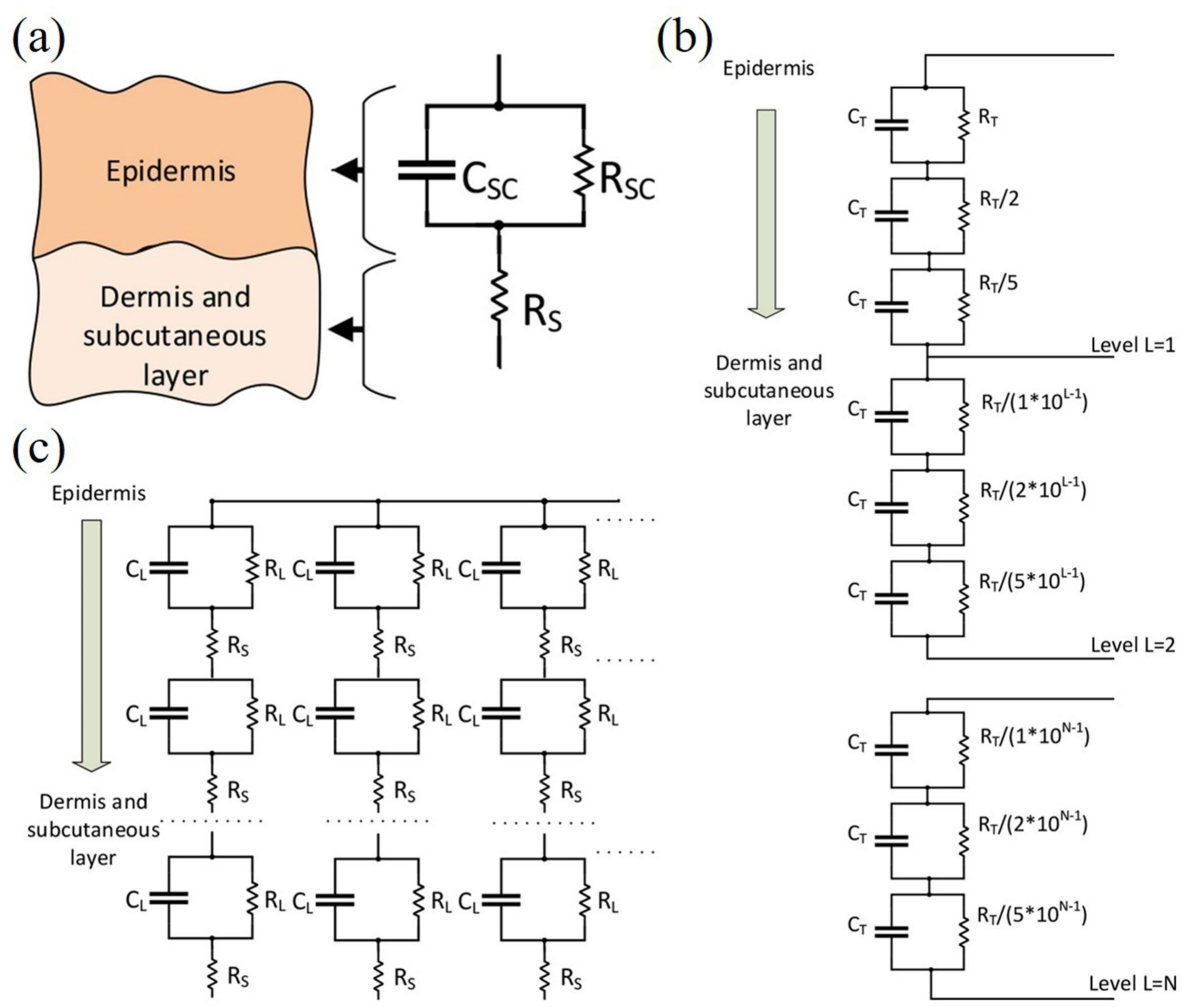
**(a)** Motague electrical model for skin impedance. **(b)** Skin impedance model provided by Tregear (1966). **(c)** Skin impedance model provided by Lykken (1970). Reproduced with permission.

Considering that the skin can be divided into several tissue layers, each of which can be divided further into sublayers. Therefore, further layering is needed on the basis of the original RC model, as shown in Figure 3. All three skin models are designed considering the hierarchical structure of the skin, i.e., the skin is considered to be a layered structure, and these layers are referred to as the extracellular medium, the intracellular medium, and the lipid bilayer. Each sublayer is represented as an RC network; therefore, the entire skin impedance model is represented as a complex cascaded RC circuit.

**Figure 4:**
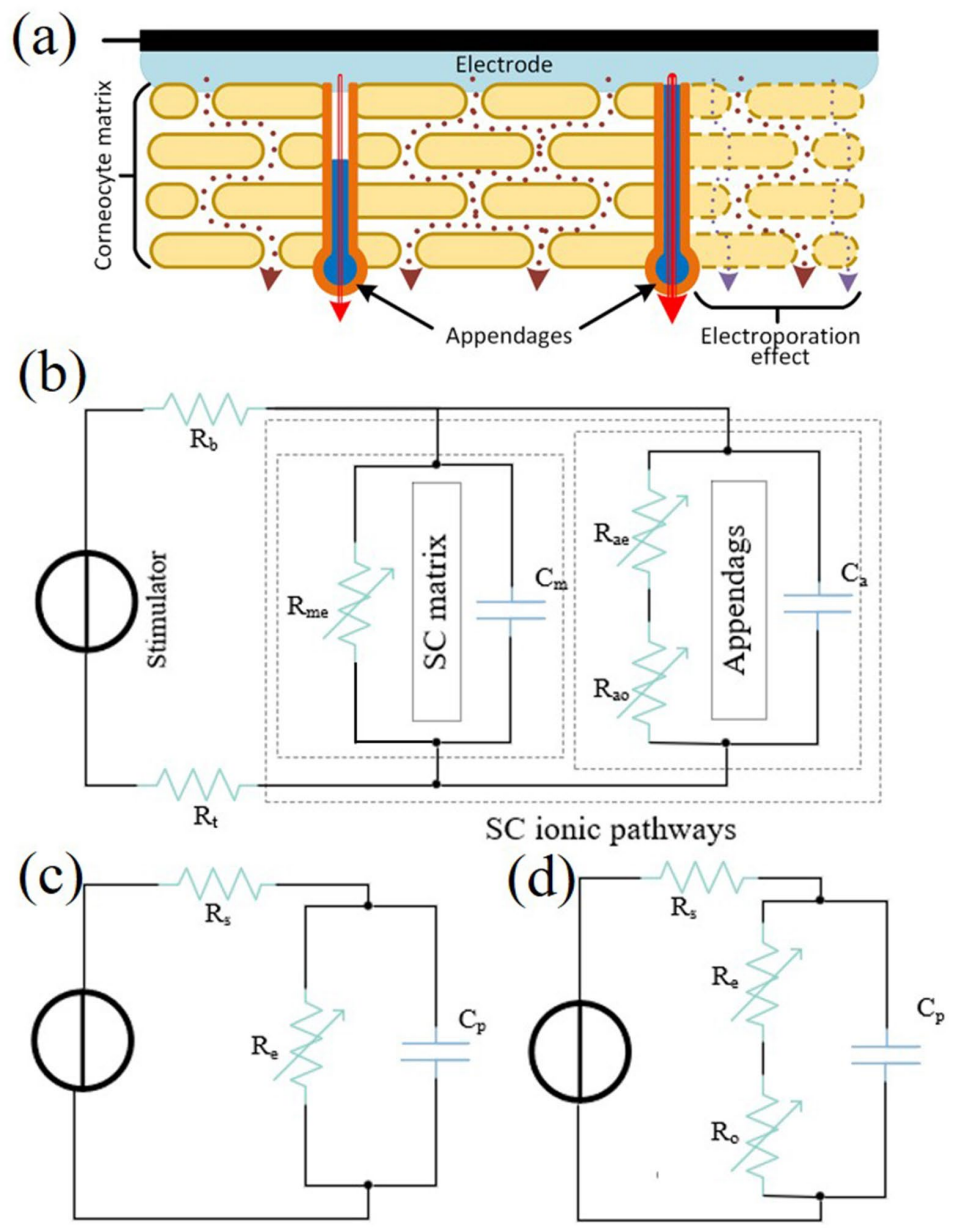
**(a)** The stratum corneum and ion channels of the skin under electrical stimulation. **(b)** A complete skin-electrode interface model taking into account the effects of electroporation and electroleakage. **(c)** A simple skin-electrode interface model with low charge pulses. **(d)** The proposed model (Vargas Luna et al., 2015). Reproduced with permission.

The skin is electrically stimulated in a process that, at the microscopic level, can be viewed as an exchange of ions between the electrodes and the skin, as shown in Figure 4a. Charged particles traverse the stratum corneum via two pathways: gaps between keratinocytes (electroporation effect) and accessory channels like sweat glands or hair follicles (electroleakage effect). As electrotactile sensation is dynamic, skin impedance modelling must account for resistive, capacitive, and time-varying properties. Johnsen et al. (2011) demonstrated that the nonlinear conductance properties of sweat glands and capillaries under the electroleakage effect could be simulated using memristor circuits. This approach significantly advanced the study of skin impedance modelling. Vargas Luna et al. (2015) investigated ionic diffusion effects of skin impedance and constructed a model for a wide range of dynamic impedance model for a wide range of stimulus intensities, as shown in Figures 4b–d. The model takes into account both electroporation and electroleakage effects, and expresses the transient and long-time effects of the skin in more detail. The model allows the predictive modelling of the current–voltage response of any pulse, leading to performance analysis and optimization of new pulse waveforms and stimulation techniques.

Overall, skin impedance exhibits complex time-varying properties, influenced by the structure of skin layers, sweat glands, and hair follicles. Recent studies have used dynamic impedance models and memristor circuits to describe the skin’s response to electrical stimulation. However, several challenges remain. The complexity of models increases computational burden, reducing efficiency in real-time applications. Additionally, individual variations in skin properties limit their generalizability. Many models are also based on specific experimental conditions, further constraining their applicability. The biocompatibility of long-term electrical stimulation also needs to be addressed. Despite these challenges, dynamic impedance models and memristor circuits strongly support the optimization of novel stimulation techniques and pulse waveforms. These advancements promote the use of electrical stimulation in tactile feedback applications. However, further optimization is required for practical implementation.

##### 3.1.2.2 Electrode skin contact model

The electrotactile sensation primarily involves a separation electrode, where the cathode and anode are separated. One electrode serves as the stimulating electrode, while the other acts as the reference electrode. The whole circuit should consider not only the impedance of the skin itself, but also the impedance of the electrode itself and the contact impedance between the electrode and the skin.

**Figure 5:**
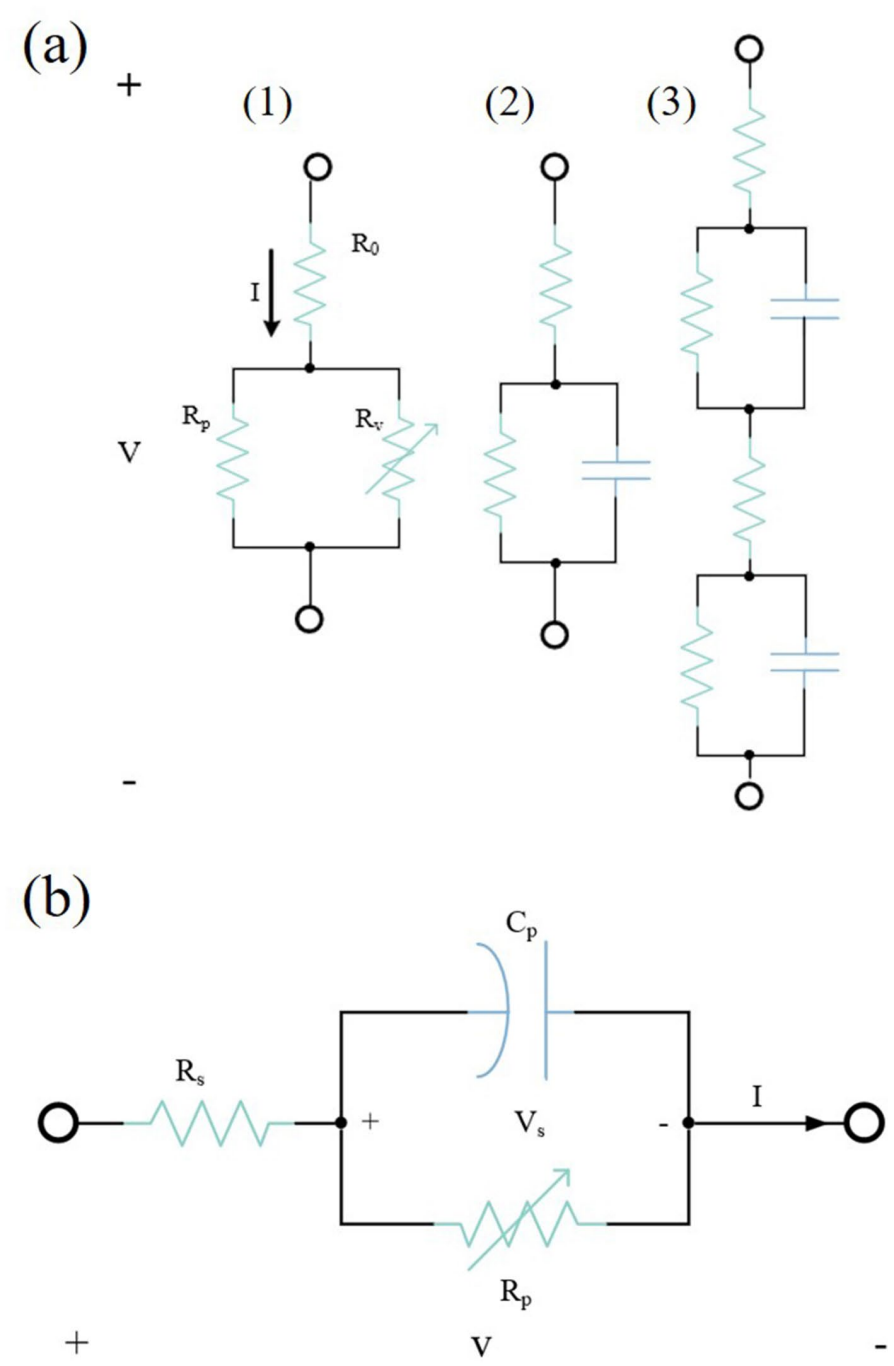
**(a)** (1) Nonlinear static electrode-skin model. (2) Single-exponential dynamic electrode-skin model. (3) Double-exponential dynamic electrode-skin model (Kaczmarek and Webster, 1989). **(b)** Cole–Cole skin-electrode circuit model, where the resistor *R*_s_ mimics the constant resistive component of the skin and deep tissues, and the variable resistor *R*_p_ and the capacitance *C*_p_ mimic all of the nonlinear historical dynamic skin impedances (Dorgan and Reilly, 1999). Reproduced with permission.

Early studies on electrode-skin contact models focused on observing changes in skin electrode resistance as the stimulation current increased. Based on these observations, mathematical models were proposed. For example, Kaczmarek and Webster (1989) designed an electrode-skin interface model as shown in Figure 5a. This model applied to low-frequency and low-current scenarios but excluded time-varying parameters. Dorgan and Reilly (1999) proposed the Cole–Cole skin-electrode circuit model for bioimpedance recognition, as shown in Figure 5b. This model captures nonlinear and time-varying changes in human skin electrical impedance under constant voltage and current stimulation. Compared with the model proposed by Kaczmarek, the model is nonlinear and time-varying as in real physiological systems, and makes a significant contribution to the study of skin electrical properties.

**Figure 6:**
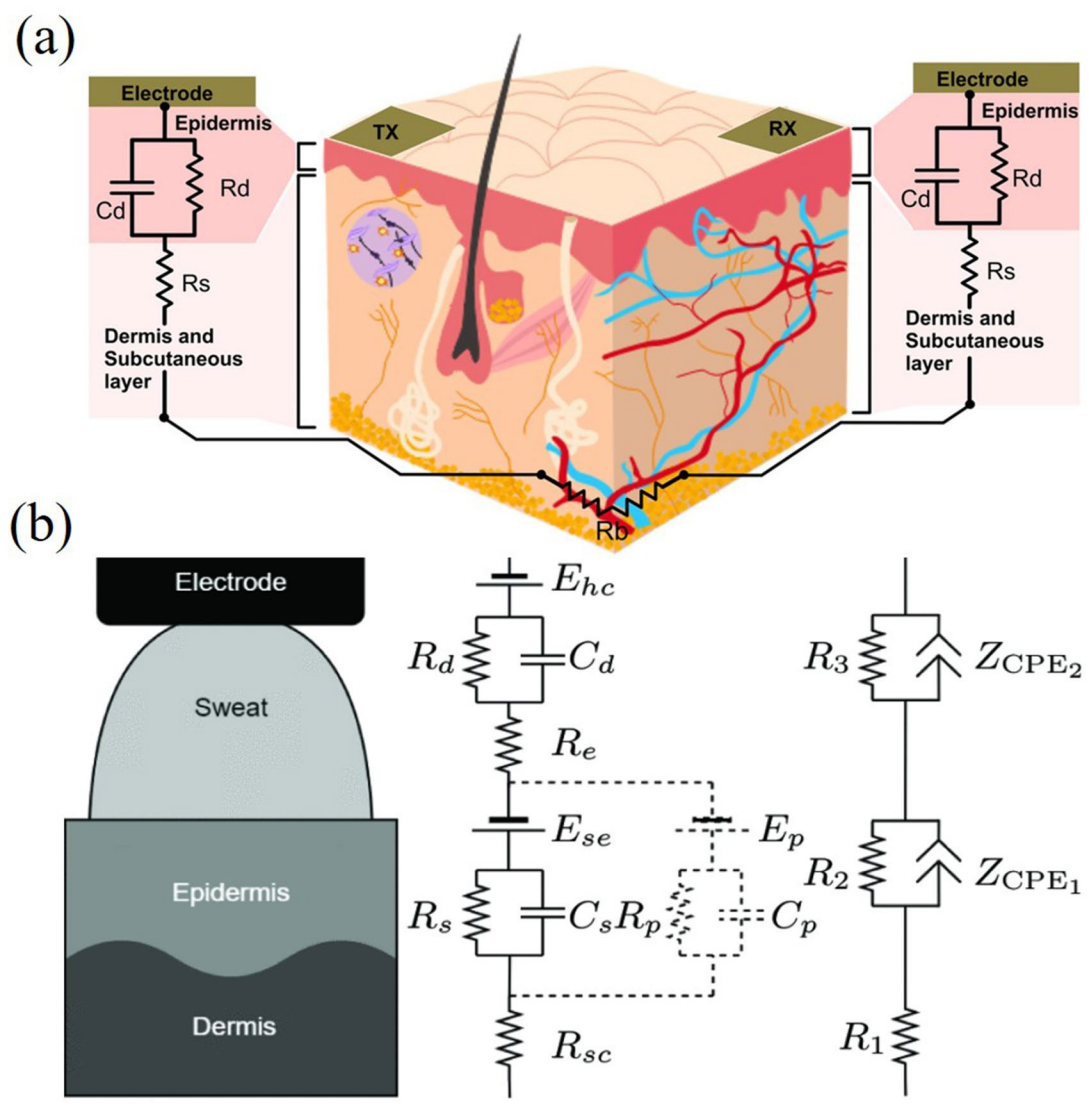
**(a)** Impedance model between Ag/AgCl electrode pairs (Peřinka et al., 2021). **(b)** Dual-parallel resistive/constant-phase element model of the electrode-skin interface on human skin (Cameron et al., 2023). Reproduced with permission.

While S. J. Dorgan’s et al. Cole–Cole skin-electrode circuit model captures many electrical properties of the skin, it can be further enhanced by incorporating a clearly defined electrode model, which requires parameterization. Saadi and Attari (2013) used electrical impedance spectroscopy (EIS) to characterise the electrical properties of the electrode-skin interface, measuring the Ag/AgCl impedance between electrode pairs and parameterized the model as shown in Figure 6a. Cameron et al. (2023) proposed a method for determining the parameter values of a double parallel resistive/constant-phase element model of the electrode-skin interface for a single Ag electrode and Ag/AgCl electrodes on human skin, as shown in Figure 6b. Initial estimation of the model parameters based on the impedance-phase characteristic data and correction of the parameter values using the least-squares method resulted in a final RMSE of 7% between the model and the experimental data. Both studies have achieved the extension of the Cole–Cole skin-electrode circuit model by parameterizing the electrode model in order to make the study of the skin electrode contact model closer to practical applications and more in line with the physiological laws during the operation of electrotactile sensation.

In conclusion, the evolution of electrode-skin interface models reflects incremental advancements in capturing the complexity of electrotactile systems. Kaczmarek’s et al. model (Figure 5a) pioneered the conceptualization of skin-electrode interactions under low-frequency, low-current conditions, offering simplicity and foundational insights. However, its exclusion of time-varying parameters and nonlinear behaviors limited its applicability to dynamic physiological systems. Dorgan’s et al. model (Figure 5b) addressed these gaps by incorporating nonlinearity and time dependence, better aligning with real-world skin impedance variations under stimulation. Yet, while it advanced physiological relevance, its lack of a parameterized electrode-specific framework hindered precise practical implementation. Saadi et al. introduced critical parameterization via EIS, explicitly characterizing Ag/AgCl electrode-skin impedance (Figure 6a), thereby enhancing model specificity for targeted applications. Nevertheless, their focus on static parameterization left room for refinement in addressing dynamic, stimulus-dependent impedance shifts. Cameron et al. advanced this further with a double parallel resistive/constant-phase element model (Figure 6b), combining robust parameter estimation via lease-squares optimization with adaptability to single-electrode configurations. While their approach improved accuracy and generalizability, the increased model complexity may pose challenges in real-time applications or scenarios requiring rapid parameter recalibration. Collectively, these models demonstrate a trajectory from conceptual simplicity to physiological fidelity and practical precision, though trade-offs persist between dynamic adaptability, parameterization granularity, and computational tractability. Future work may focus on hybrid models that balance Cameron’s accuracy with Dorgan’s dynamic nonlinearity while retaining Saadi’s electrode-specific parameterization.

##### 3.1.2.3 Neuronal conduction models

In the study of neuronal conduction models, early foundational work established mathematical frameworks to describe the electrical current conduction on neurons, they linked membrane potentials to transverse membrane currents, describing the propagation action of action potentials along the axon (Fitzhugh, 1962; Hodgkin and Huxley, 1990; Rushton, 1951). McNeal (1976) proposed a model for the electrical properties of myelinated nerves, building on the work of Fitzhugh and Hodgkin. This model ignored the effect of nerve fibres on the electric field and calculated nerve fibre thresholds for finite-duration pulses using non-contact electrodes. Additionally, it considered the impact of nerve fibre diameter on activation thresholds (Vargas Luna et al., 2015). Ratta (1999) developed a model similar to that of McNeal, proposing the generalised activation function theory. This theory challenged the assumption that neural excitability depends on negative current density or extracellular voltage strength (Hodgkin and Huxley, 1990). While these models captured core electrophysiological principles, their reliance on idealized scenarios and static parameters limited their ability to simulate real-world physical phenomena, such as tissue impedance effects or spatially distributed electric fields. Computational constraints further restricted dynamic, multiscale simulations of neuronal behavior.

With the development of computer science, the advent of computational tools like COMSOL Multiphysics and NEURON revolutionized the study of neuronal conduction by enabling multiscale, Multiphysics simulations. COMSOL excels in modelling macroscopic tissue-electrode interactions, such as electric field distributions and current diffusion depths. Kuhn et al. (2009) used COMSOL to perform hierarchical finite element modelling of the arm and used NEURON for neuronal modelling. They analyzed how array electrode gaps and gel resistivity affect nerve activation. Zhu et al. (2015) used COMSOL for finite element modelling of the arm and simulated it with the help of neuron model, and explored the effect of the thickness and resistivity of the gel and cuticle on the tactile feedback thresholds using the activation function as an indicator (Fitzhugh, 1962; Ratta, 1999). However, COMSOL’s limited granularity in modelling single-neuron dynamics necessitates integration with specialized neuronal simulators. In contrast, NEURON focuses on microscale electrophysiology, employing Hodgkin–Huxley formalism to simulate ion channel dynamics and action potential propagation. Therefore, in 2017, 2019, and 2020, the Sui’s team used NEURON to build more fine and complex neuron models, to study the variation of threshold current with different fibre diameters and different stimulation electrode sizes, and explored the relationship between nerve fibre diameters and sensory quality, etc. (Zhu et al., 2017; Ye et al., 2019; Ge et al., 2020).

While NEURON provides unparalleled resolution of neuronal behavior, its abstraction of tissue-level properties hinders direct translation to engineering parameters like electrode placement or stimulation waveforms. Hybrid approaches have emerged to bridge these scales. In 2019, Gloria similarly combined the COMSOL finite element model and the neuronal model proposed by McNeal (1976) to model the finger and investigate the dependence of transcutaneous electrotactile stimulation on electrode layout and excitation pattern. In addition to the above studies combining tissue and neuronal models, others have focused on the effects induced by the current in human tissues, such as the depth of diffusion and the temperature rise coefficient (Medina and Grill, 2015; Soltanzadeh et al., 2020).

**Figure 7:**
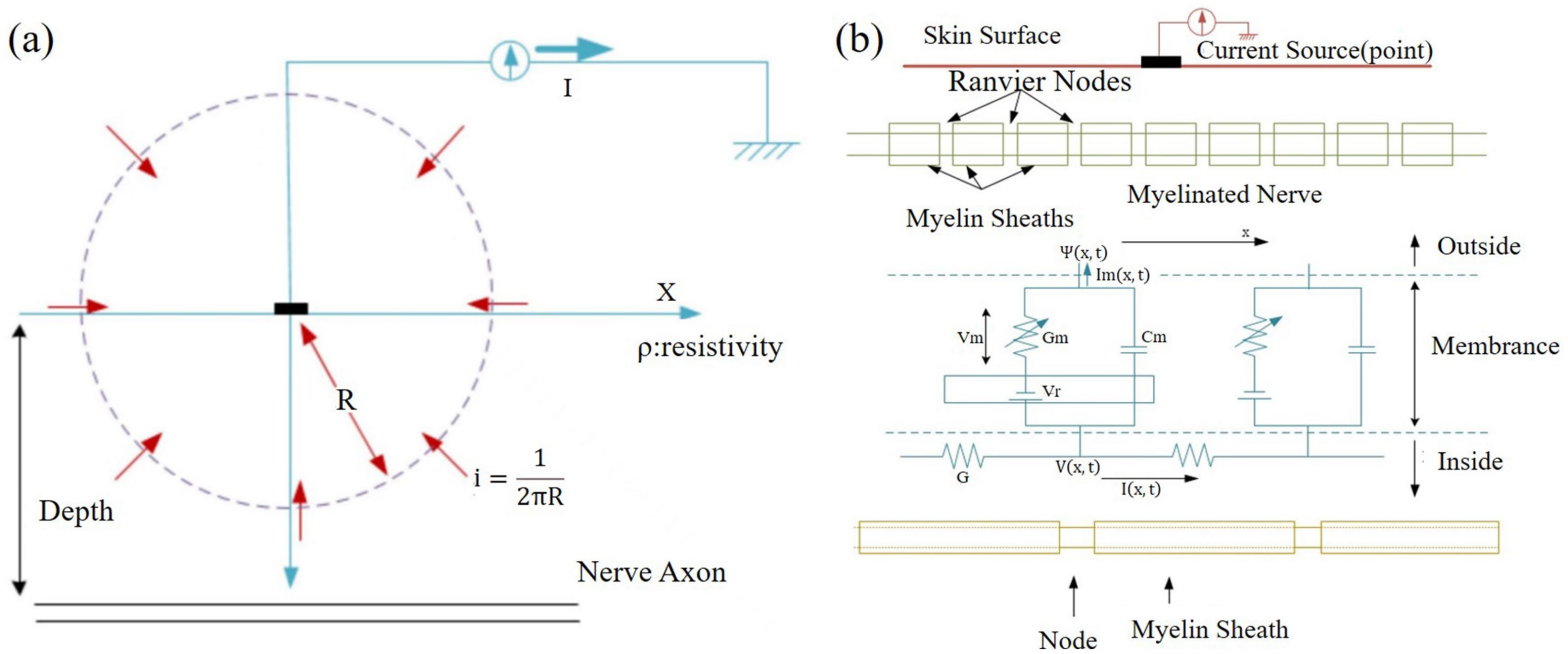
**(a)** Current stimulation at the skin surface. **(b)** Cross-section and equivalent electrical circuit of a nerve axon electrically stimulated from the skin surface (Kajimoto et al., 1999). Reproduced with permission.

Neuronal conduction modelling studies also involve the conduction of electrical current stimuli through the skin, acting on tactile receptors inside the skin and triggering the physical process of generating nerve impulses. By modelling, simulating and varying the stimulation parameters, it is investigated how to stimulate the mechanoreceptors to obtain a suitable virtual tactile effect. For example, Kajimoto et al. (1999) proposed activation functions similar to those of Ratta (1999), as shown in Figure 7. And based on this, they proposed the use of appropriately weighted array electrodes or the use of anodic current stimulation and cathodic current to stimulate three kinds of mechanoreceptors in human skin, respectively. Fei et al. (2014) derived the excitation function for nerve cell stimulation using a subcutaneous nerve model. They activated different mechanoreceptors on the array, including Meissner’s corpuscles (RA), Merkel’s cells (SAI), and Pacinian’s corpuscles (PC), by adjusting current parameters. He et al. (2016) designed a variety of stimulation methods and finally found the optimal stimulation methods for two mechanoreceptors (Meissner corpuscles and Merkel cells). The study of these stimulation methods for tactile receptors provides solid theoretical support for further improvement of virtual tactile reproduction effects.

In conclusion, the progression from early mathematical abstractions to advanced multiphysics simulations reflects a paradigm shift in neuronal conduction research. While mathematical models laid the groundwork for understanding neural excitability, tools like COMSOL and NEURON have unlocked unprecedented resolution in both tissue- and neuron-scale phenomena. Future work must further integrate these approaches to optimize electrotactile interfaces, balancing computational efficiency with biological fidelity to advance tactile feedback technologies.

#### 3.1.3 Psychophysical studies of electrotactile perception

Psychophysical methods play a key role in electrotactile research. They are primarily used to investigate the relationship between external physical stimuli and tactile perception. Current psychophysical approaches place a major emphasis on the use of non-invasive means to study different sensory modalities and biological sensory mechanisms. The advancement of signal detection theory has enabled researchers to design robust experiments. By integrating statistical models, they can describe psychological activities such as decision tendencies, preferences, and response biases during tactile perception.

Compared with subjective physiological and psychological factors, the effects of physical factors on electrotactile perception are easier to quantify and thus more suitable for research. Stimulation mode covers factors like contact frequency and duration. Electrode characteristics include material, shape, and size (contact area). Power source factors encompass waveform, frequency, and strength. Due to the strong coupling between these factors, the effect of a single factor can be quantified, but the combined effect of multiple factors is more difficult to describe. Therefore, researchers have mostly used psychophysical experimental means to examine these factors qualitatively or semi-quantitatively, and to analyze and assess the relationship between these factors and electrotactile perception by detecting threshold values at different levels.

**Figure 8:**
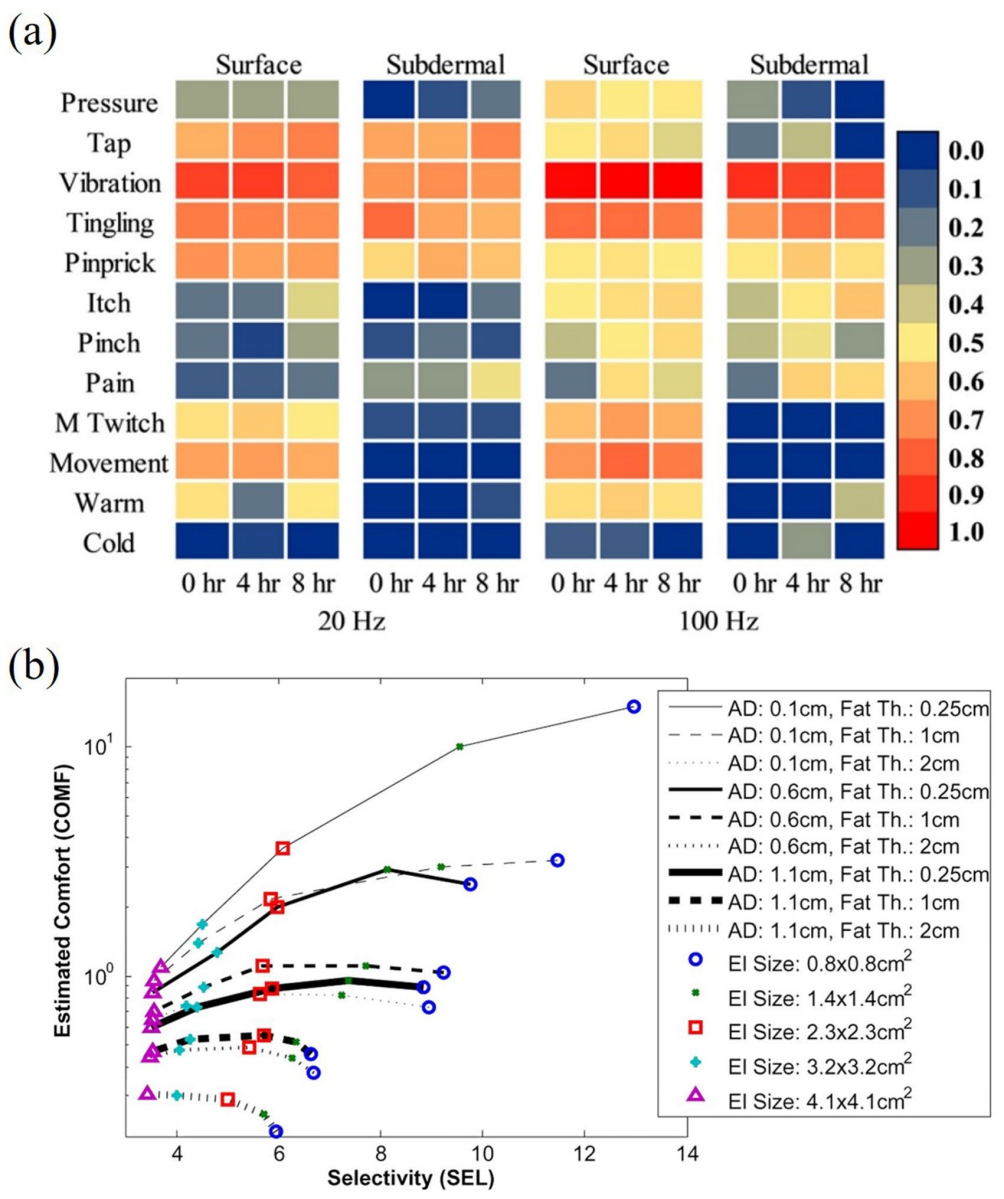
**(a)** Comparison of sensory quality between epidermal and subcutaneous stimuli (Dong et al., 2020). **(b)** Comfort and selectivity for different electrode sizes for different fat thicknesses and stimulation depths (Kuhn et al., 2010). Reproduced with permission. Reproduced with permission.

The main stimulus modalities that affect electrotactile perception include contact pressure, contact frequency and contact duration. For example, Tashiro and Higashiyama (1981) investigated the relationship between pulse intensity and pulse duration and sensory quality. Buma et al. (2007) explored the effect of inter-stimulus interval on the adaptation to electrotactile stimulation. Warren (2009) found that electrotactile stimulation with a certain gesture produces the skin hare touch illusion, which is the illusion that the user will feel the skin at a non-stimulated point. i.e., the user would feel the hopping phenomenon at non-stimulated points. Chen et al. (2019) investigated the effect of different skin electrode contact conditions on the stimulation current pain threshold, and found that the stimulation current pain threshold would be reduced under non-stable contact conditions. Dong et al. (2020) compared the sensory quality of epidermal stimulation and subcutaneous stimulation, as shown in Figure 8a. Collectively, these studies demonstrate the significant influence of electrotactile stimulation modalities on feedback quality. Optimizing these modalities is crucial for advancing electrotactile technology, expanding its applications, enhancing safety and comfort, and fostering interdisciplinary research.

Stimulation electrodes that affect electrotactile sensation include electrode material, electrode shape, and electrode size (the contact area between the electrode and the skin). Brummer and Turner (1975) investigated electrode material and found that precious metal or conductive polymer electrodes could reduce unnecessary electrochemical reactions, thus improving the safety and effectiveness of electrical stimulation. Kuhn et al. (2010) used finite element modelling to assess the influence of different electrode sizes on selectivity and perceived comfort. The effects of different electrode sizes on selectivity and perceived comfort, as shown in Figure 8b. This study showed that the optimization of electrode size can significantly improve user comfort and stimulus selectivity, thus improving the quality of the overall tactile experience. Mørch et al. (2011) evaluated the perceptual thresholds of electrodes of different sizes on the palmar side of the forearm of healthy volunteers using an adaptive two-alternative forced-choice algorithm. The study examined the effect of electrode size on the excitatory values of different nerve fibres. Wang et al. (2013) found that when the electrode pair spacing was larger, the current required to produce the same sensation was smaller. This finding suggests that optimizing electrode spacing can maintain efficient tactile stimulation effects while reducing energy consumption. In summary, these studies collectively reveal the importance of factors such as the material, size and spacing of the electrodes for electrotactile feedback. By optimizing these parameters, the efficiency, comfort and accuracy of electrically stimulated tactiles can be significantly enhanced, thereby promoting the development and application of virtual tactiles.

**Figure 9:**
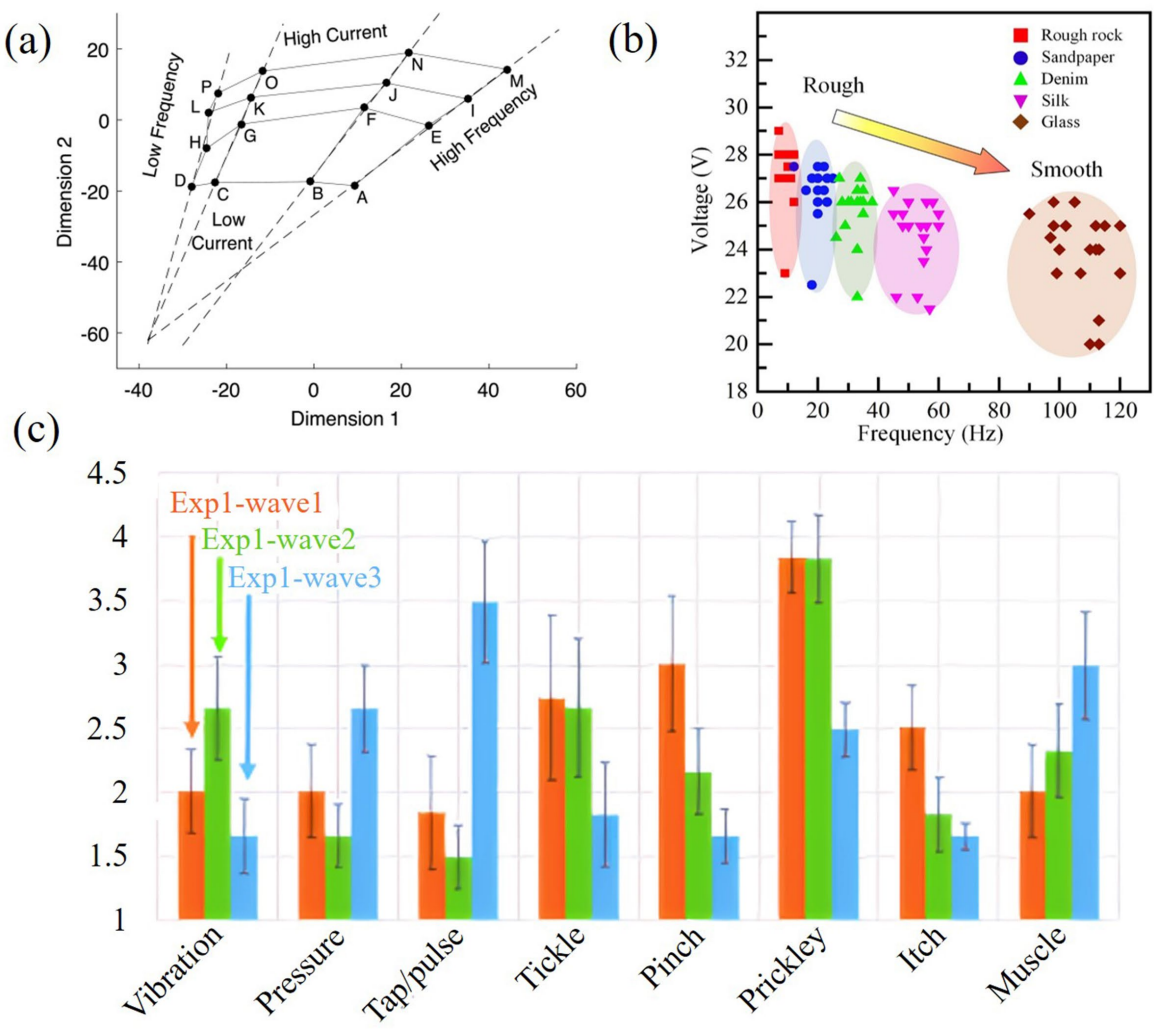
**(a)** Relationships between two perceptual dimensions and pulse amplitude with pulse frequency (Kaczmarek et al., 2017). **(b)** Relationship between pulse width-frequency and sensory roughness (Lin et al., 2022). **(c)** The effect of different waveforms on the intensity of different sensory qualities (Parsnejad et al., 2019). Reproduced with permission. Reproduced with permission.

Stimulus parameter that affects electrotactile perception refers to power factors such as power waveform, power frequency, and power intensity. Geng et al. (2012) evaluated the effects of electrical stimulation site, number of pulses, number of stimulation channels (single versus dual), and interleaving time between the two channels on the quality of sensation evoked. Wang et al. (2013) investigated the effects of pulse parameters such as amplitude, pulse width and frequency of pulse parameters on subjective intensity and sensory quality. Kaczmarek et al. (2017) concluded that electrotactile perception consists of two perceptual dimensions, perceptual frequency and perceptual intensity. They further studied how pulse amplitude and frequency relate to these dimensions, as shown in Figure 9a, as the amplitude and frequency of the pulse decrease, the values of these two dimensions will also decrease. Vardar et al. (2017) found, through psychophysical experiments, that the frequency dependence of the electrical properties of human skin and tactile sensitivity led to sensory differences in low-frequency waveforms. Parsnejad et al. (2019) investigated the qualitative effects of different waveforms on different sensory qualities using coplanar electrodes, as shown in Figure 9c, different waveforms will lead to different perceptual quality in different perceptual forms. Lin et al. (2022) investigated the relationship between pulse voltage-frequency and sensory roughness, as shown in Figure 9b, with the decrease of voltage and the increase of pulse frequency, the perception of roughness changes from rough to smooth. Zhou et al. (2022) proposed an improved measurement method for the psychophysical measurements of detection threshold (DT), pain threshold (PT), minimal perceptible difference (JND), parametric intensity property (PIP), and sensitivity index (SI). They further analyzed how pulse amplitude relates to DT and PT, pulse frequency to JND, and pulse amplitude and width to sensation intensity.

Through the above research, the influence mechanism of current parameters on electrotactile perception can be obtained. Studies demonstrate that current amplitude directly determines perceptual intensity, with higher amplitudes enhancing subjective tactile strength but requiring careful balancing between DT and PT to avoid discomfort. Frequency influences perception through dual mechanisms: high-frequency stimuli (>50 Hz) tend to evoke smoother sensations, while low-frequency ranges (<50 Hz) may induce “rough” or “vibratory” qualities due to frequency-dependent skin impedance. Frequency also correlates with the JND, necessitating precise tuning to optimize perceptual discrimination. Pulse width interacts nonlinearly with amplitude and frequency: wider pulses (e.g., >200 μs) enhance perceived intensity, but their combination with frequency (e.g., high-frequency narrow pulses vs. low-frequency wide pulses) significantly shapes spatiotemporal tactile attributes, such as roughness or continuity. Furthermore, spatiotemporal modulation of multi-channel parameters (e.g., interleaving time) expands the dimensionality of tactile encoding. Crucially, these parameters do not act in isolation; instead, they interact through the skin’s frequency-varying electrical properties (e.g., impedance) and neural encoding mechanisms (e.g., activation function thresholds), forming a multidimensional perceptual space. Optimizing electrotactile technology requires integrating psychophysical metrics (e.g., DT, PT, and JND) with coupled parameter models (e.g., amplitude-frequency-pulse width response surfaces) to balance perceptual strength, quality, and safety, ultimately enabling user-customizable high-fidelity tactile feedback.

In conclusion, these studies provide valuable insights into how different stimulus power factors shape electrotactile perception. They offer a theoretical and experimental foundation for optimizing electrotactile technology. This knowledge enables developers to fine-tune stimulus parameters, enhancing both the technology’s performance and user experience.

After a large number of studies on the influencing factors of electrotactile sensation, researchers began to focus on how to enhance the perceived quality and comfort of electrotactile sensation. For example, Gregory et al. (2009) developed a customised electrotactile display terminal for data acquisition and an individual bioimpedance parameter identification method based on the Cole–Cole skin-electrode circuit model. Using this approach, the parameters of the skin-electrode interface circuit model were characterized. Psychophysical experiments confirmed that the method enhanced the consistency and comfort of tactile rendering. D’Alonzo et al. (2014) experimentally verified that a hybrid physical vibration-electro-tactile (HyVE) stimulation modality could effectively increase the resolution of tactile feedback, thereby improving the tactile feedback perceptual quality. Rahimi et al. (2019) found that the mechanoreceptors would be fatigued when subjected to electrical. To address this, they developed a system that adjusted microcurrent parameters to stabilize tactile rendering. This approach ensured consistent tactile feedback for different individuals and varying locations on the same individual. Yang et al. (2020) proposed a multi-channel transcutaneous electrical nerve stimulator with online skin impedance measurement. Through psychophysical experiments on subjects, it was verified that the method could adaptively adjust the stimulation intensity according to the change of impedance, thus improving the safety and applicability of the electrotactile feedback system.

These studies mentioned above have made significant progress in improving the quality and comfort of electrotactile feedback, but there are some limitations or shortcomings.

Firstly, the generalizability of the models and methods, most of the current studies are based on specific experimental groups or individuals, and there are significant differences in skin properties and neural responses among different populations, so the methods mentioned above may have limitations in terms of generalization. At the same time, environmental factors such as temperature, humidity, etc. have a significant effect on the electrical properties of the skin and neural responses, and the above studies may not be comprehensive enough in controlling these environmental variables, which may lead to pure bias of the results in practical applications.

Secondly, there is the complexity of the technical implementation, which can be divided into two types: the limitations of hardware devices, and the difficulty of algorithm optimization. The limitation of hardware devices such as the Cole–Cole model-based electrotactile display terminal developed by Gregory et al. may suffer from excessive hardware implementation complexity and high cost, which is not conducive to large-scale promotion and application. Algorithm optimization difficulties such as the online skin impedance measurement function proposed by Yang et al. require complex algorithmic support, and these algorithms are difficult to optimize and debug, and may be interfered with by a variety of factors in practical applications.

Finally, the inherent limitations of psychophysical experiments, psychophysical experiments mainly rely on the subjective feelings of the subjects, and this subjectivity may lead to bias in the experimental results. At the same time, there may be differences in feelings between different subjects and between the same subject at different points in time, which increases the difficulty of reproducibility of experimental results. These findings collectively highlight the complexity of electrotactile sensation modulation and emphasize the need for standardization in psychophysical protocols to ensure consistent cross-study comparability. Moreover, understanding how different stimulation factors interact to affect perceptual outcomes is crucial for guiding the design of adaptive feedback systems.

### 3.2 Virtual tactile technology and its equipment

#### 3.2.1 Wearable non-invasive electrode arrays

Wearable non-invasive electrodes are mainly composed of two parts: the stimulating electrode and the grounding electrode. When current is applied to the stimulation site to produce tactile sensation, the current will flow from the positive electrode to the negative electrode through the body. The position of the grounding electrode, as the negative electrode, affects the position, density, depth, and degree of diffusion of the entire current pathway. Since both parts are in direct contact with human skin in the process of providing tactile feedback, the selection of electrode materials and the electrode settings for the tactile feedback modality of electrical stimulation have become an important criterion in addition to indicators such as positioning accuracy and induced sensory intensity. The common electrode settings in the literature can be divided into concentric electrodes and separated electrodes, and the common separated electrode settings applied to the finger area can be subdivided into transducer dorsum, phalangeal ring, palmar, and dorsum of the finger, and so on (Yoshimoto et al., 2015; Tezuka et al., 2016).

Table 1 describes in detail the advantages and disadvantages of different placement positions of grounding electrodes under the separated electrode setup for finger application scenarios. Further, Stephens-Fripp et al. (2020) compared the various performance metrics of concentric and separated electrodes through psychophysical experiments, and the results showed that concentric electrodes can reduce the generation of discomfort (e.g., pins and needles, pinches, or pains), and thus concentric electrodes may become the main form of future research.

**Table 1:** Advantages and disadvantages of different grounding electrode types.

Style	Advantages	Disadvantages
Finger rings (Yoshimoto et al., 2015)	Small amplitude of stimulation current required to produce sensation at the fingertip of the finger	Current stimulation is produced on the inside of the finger and requires five grounded electrodes
Trans dorsal (Yem et al., 2018)	Stimulation electrodes on five fingers can share the same grounding electrode	Current stimulation occurs on the inside of the fingers as well as on the palm of the hand
Palm (Rahimi et al., 2019)	Stimulation electrodes on all five fingers can share the same ground electrode	Current stimulation is produced on the inside of the fingers and on the palm of the hand
Dorsum of the fingers (Tezuka et al., 2016)	Current and sensory points are concentrated on the stimulated part of the finger	To achieve tactile feedback on all five fingers, five ground electrodes are required

**Figure 10:**
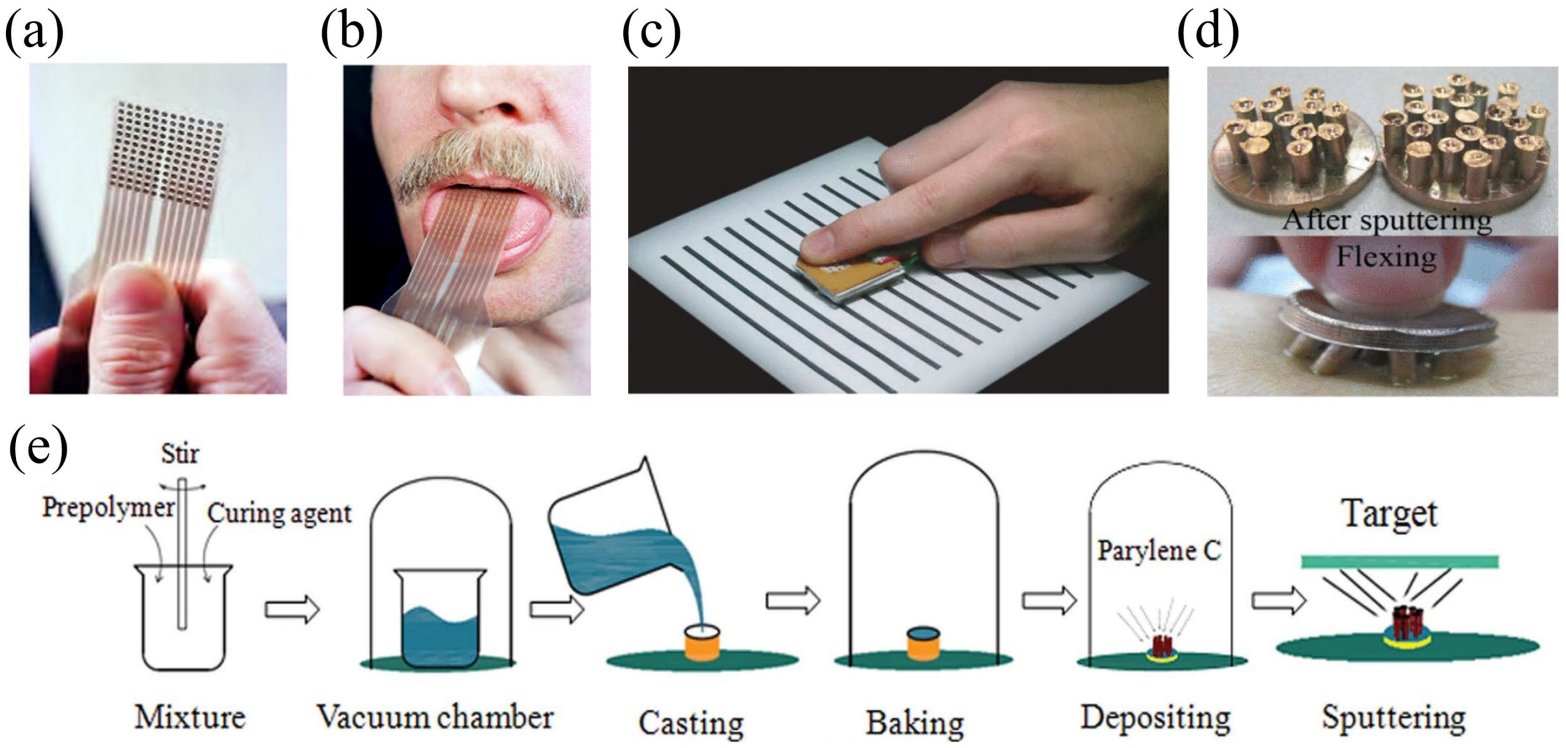
**(a,b)** Photo of tongue display unit electrode array (Kaczmarek, 2011). **(c)** SmartTouch prototype system in which optical sensors capture a visual image (black and white stripes) and display it through electrical stimulation (Kajimoto et al., 2004). **(d)** Prototype of PDMS-based flexible dry electrode after sputtering and flexing (Wang et al., 2012). **(e)** Schematic of the fabrication process of the PDMS-based flexible dry electrode (Kaczmarek et al., 1991). Reproduced with permission.

Conventional rigid printed circuit boards (PCBs) are often replaced by flexible printed circuit boards (FPCBs) in electro-tactile stimulation applications because they do not fit well on the surface of human skin. These flexible circuits are widely used in wearable devices such as gloves or motion-capture suits to ensure flexibility and comfort for real-world applications. These electrode materials are usually derived from those used in electrophysiological recordings, such as electrocardiograms (ECGs), electromyograms (EMGs) and electroencephalograms (EEGs) (Jung et al., 2020). In practice, electrode materials can be categorised into dry electrodes and adhesive gel electrodes. Dry electrode material clad with mixed layers of metals such as gold, stainless steel, platinum, etc., or certain polymers, etc. (Kaczmarek et al., 1991; Kaczmarek, 2000). Kaczmarek proposed a tongue display device (TDU) to provide electrotactile stimulation on the tongue via surface electrodes, with a gold plating layer of the electrodes used to minimise electrochemical reactions on the tongue, as shown in Figures 10a–c. However, with prolonged use (hundreds of hours), the gold layer may gradually degrade and affect the performance of the device. In addition, the comfort of electrotactile sensation is affected by the electrode geometry, skin condition, and stimulation waveform (Kajimoto et al., 2004; Kaczmarek, 2011). Wang et al. (2012) proposed new flexible dry electrode for long term electroencephalography measurements. This electrode array is made of polydimethylsiloxane which is made of polydimethylsiloxane and is expected to expand to the application of electrotactile stimulation, as shown in Figures 10d,e.

**Figure 11:**
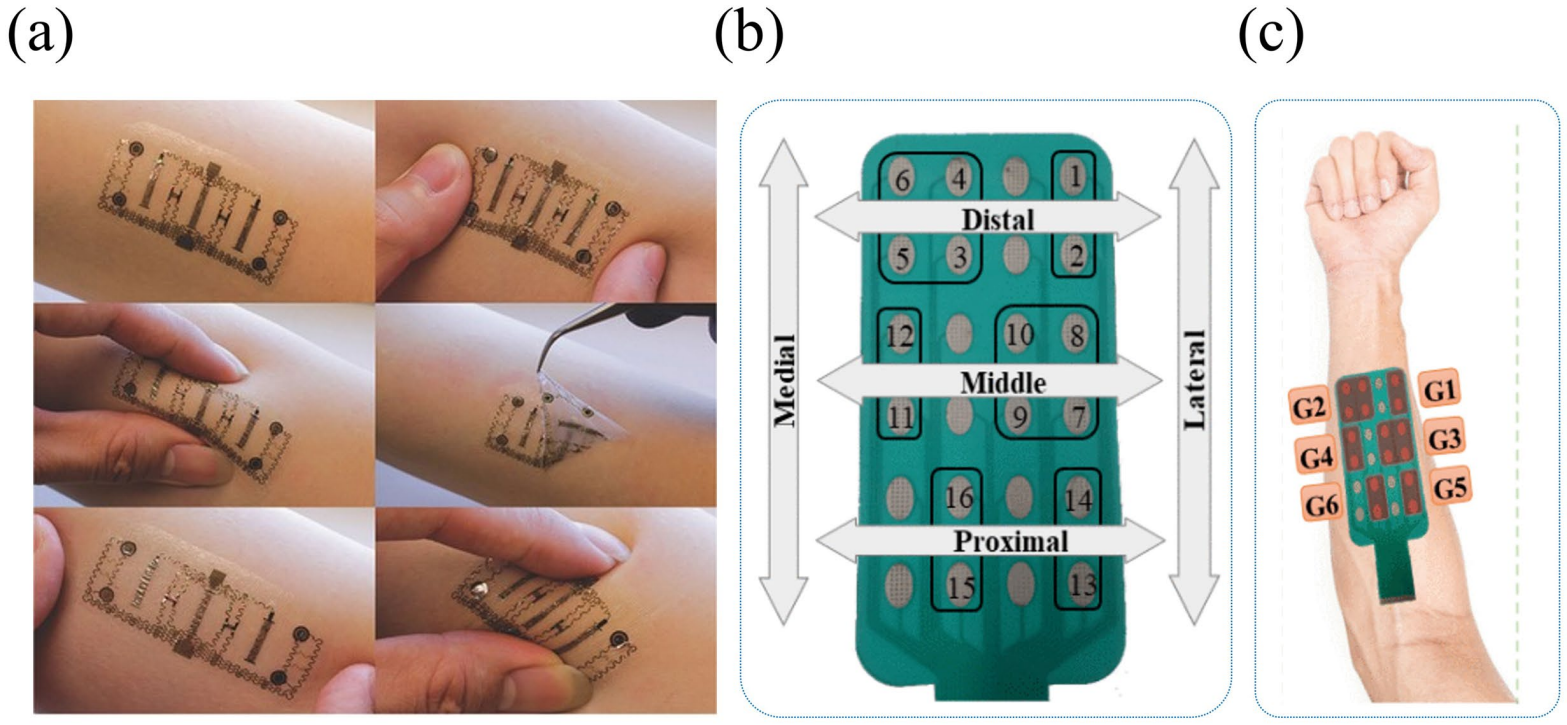
**(a)** The design of an ultrathin, conformal electronic device (Xu et al., 2016). **(b,c)** A biocompatible flexible matrix electrode which was made of a polyester layer, Ag/AgCl conductive layer, and an insulation coating covering the conductive leads (Abbass et al., 2021). Reproduced with permission.

However, both dry and gel electrodes face the problem of contact instability in long-term applications. For example, in dry electrodes, the contact area between the electrode and the skin may change with hand movements, leading to impedance fluctuations, which in turn affects the stability of tactile feedback. To address this problem, Abbass et al. (2021) proposed a tactile feedback system based on flexible matrix electrodes to improve the electrode-skin contact by covering the electrodes with a conductive biocompatible hydrogel, as shown in Figures 11b,c. However, with the prolongation of the usage time, the adhesive gel electrodes may still suffer from poor contact, which affects their performance. In response, Xu et al. (2016) developed an integrated ultrathin conformal electronic platform made of silicone elastomer, which can provide more stable electrical stimulation, as shown in Figure 11a.

Although gel electrodes still dominate the field of electrostimulation, new flexible fabric electrodes offer new directions for future development. Wearable electrostimulation devices with flexible fabric electrode arrays can generate virtual haptics through silk-based electrodes, and the silk substrate, due to its high flexibility, stretchability, and breathability, allows the electrohaptic device to maintain natural tactile feedback without interfering with the fine movements of the hand, which greatly improves the wearing comfort (Cao et al., 2024). The close contact characteristics of this new fabric electrode can solve the problem of unstable contact of traditional electrodes during hand movements, thus ensuring a consistent tactile feedback experience and promoting the application and development of electro-stimulation technology in haptic feedback devices.

#### 3.2.2 Electrotactile modality

There are two modes for electrical stimulation: current control and voltage control. They both directly act on human skin or nerves, and the resulting electrical stimulation can activate the tactile receptors under the skin, thus triggering the brain to process the incoming stimulation signals and produce corresponding tactile sensations. The effect of electrotactile feedback is not only related to the design of the electrode array and the location of the grounded electrodes as mentioned in the previous section, but is also influenced by a series of stimulation signal parameters. Stimulus signals are usually delivered in the form of electrical pulses, which can be categorised as constant voltage source stimulation and constant current source stimulation. The stimulation current applied to the human body from a constant voltage source can fluctuate dramatically due to individual variability, the actuator-body interaction interface, and the instability of the impedance of the electrical stimulation circuit. The tactile perception of the human body is closely related to the current size, and the current fluctuation will greatly affect the human body’s tactile perception, so the constant-current source stimulation is more effective. In the microcurrent-based tactile feedback modal research, the main focus on the current amplitude, frequency and pulse width of the three parameters on the tactile feedback. As shown in Table 2, researchers have determined some relationships between human tactile perception and current parameters through a large number of experiments: Fei et al. (2014) achieved tactile feedback of vibration sensation and pressure sensation of different intensities by modulating the pulse width and frequency of the current mode; Djozic et al. (2015) investigated the relationship between current frequency, pulse width and human tactile perception, and found that there was no significant difference in human tactile perception ability under different current pulse widths. Alotaibi et al. (2020) found that the effect of current amplitude on tactile perception was greater than that of pulse width. Butikofer and Lawrence (1979) suggested that when the current pulse width exceeds 500 μs, a tingling sensation is produced on the surface of human skin.

**Table 2:** Current stimulation signal parameters in the relevant literature.

References	Current amplitude/mA	Frequency/Hz	Positive pulse width/μs	Tactile sensation
Yem et al. (2018)	0–5	20–50	100	Softness and tack strength
Araiza Illan et al. (2019)	−5 to 5	100	450	Vibration sensation and pressure sensation
Fei et al. (2014)	1–3	0–1,000	10–500	Vibration and pressure sensation
Yoshimoto et al. (2015)	0.9–3	0–100	200	Roughness of real materials
Kaczmarek and Haase (2003)	0–6.5	20–40	68.03	Different patterns
Kajimoto et al. (1999)	0–2	0–1,000	200	Sense of vibration and pressure
Sato and Tachi (2010)	0–5	0–210	20	Distribution of force vectors
Withana et al. (2018)	1–10	0–100	200–1,000	Different patterns

Overall, the current amplitude affects the strength of tactile feedback; when the stimulus signal amplitude is less than the sensory threshold, the human body cannot feel tactile feedback; when the stimulus signal amplitude is greater than the pain threshold, the human body will feel a tingling sensation. There is no significant difference in the human tactile perception ability under different current pulse widths, but when the current pulse width exceeds 500 μs, a tingling sensation will be produced on the surface of human skin. Frequency not only affects the strength of tactile feedback, but also affects the type of tactile feedback, for example, low-frequency stimulation signals can produce pressure sensations, and high-frequency stimulation signals can produce vibration sensations.

#### 3.2.3 Virtual tactile rendering

Virtual tactile rendering refers to the process of generating tactile feedback through tactile devices and algorithms. This method enables users to have a tactile experience in interacting with virtual or real objects. Conventional prostheses lack tactile feedback, and this lack of sensation interrupts the sensory closure loop between the brain and the hand, leading to the abandonment of the prosthetic hand by the residual limb user. Therefore, early virtual tactile research focused on restoring tactile feedback to amputees through sensory substitution (Bach-y-Rita and Kercel, 2003). With the advancement of technology, the application scenarios of virtual tactile sensation have gradually expanded to the fields of virtual reality and teleoperation, aiming to provide users with more realistic tactile sensations.

Tactile feedback techniques can be divided into two main categories: invasive and non-invasive. Invasive feedback provides lower stimulation thresholds by implanting electrodes into the nervous system in direct contact with afferent nerves. For example, Raspopovic et al. (2014) were able to decode different grasping tasks in real time by stimulating the median and ulnar nerves with transverse multichannel intra-bundle electrodes, helping amputees to obtain tactile feedback and improve prosthetic control. However, invasive electrodes still have limitations in terms of durability and neural interface technology, which have affected their diffusion in practical applications (Fallahian et al., 2016). In contrast, non-invasive feedback is more widely used due to its non-invasive nature, especially in the fields of virtual reality, teleoperation and texture communication (Kitagawa et al., 2005; Pamungkas and Ward, 2016; Altinsoy and Merchel, 2012). There are two main modalities of non-invasive feedback: vibrotactile and electrotactile. Vibrotactile transmits physical forces mechanically, while electrotactile stimulates the neural afferent system in the skin through electrodes. Vibrotactile was early used in prosthetic control, such as the force feedback system developed by Pylatiuk et al. (2006) which feeds back grip force information through vibrating motors to enable more precise gripping of prosthetic limbs.

**Figure 12:**
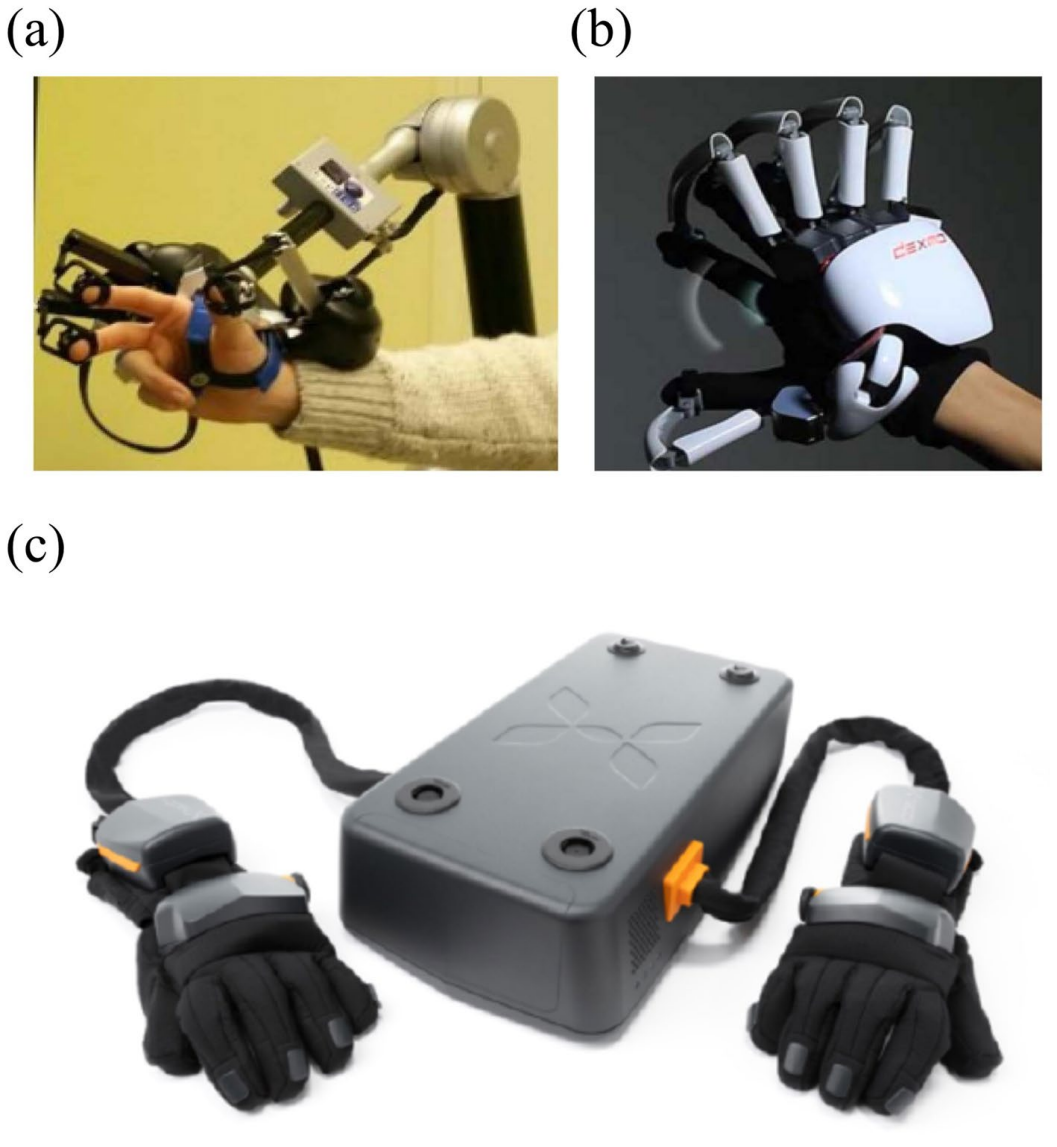
**(a)** H-glove (Perret et al., 2017). **(b)** Dexmo glove. **(c)** HaptX glove (Varga, 2018). Reproduced with permission.

**Figure 13:**
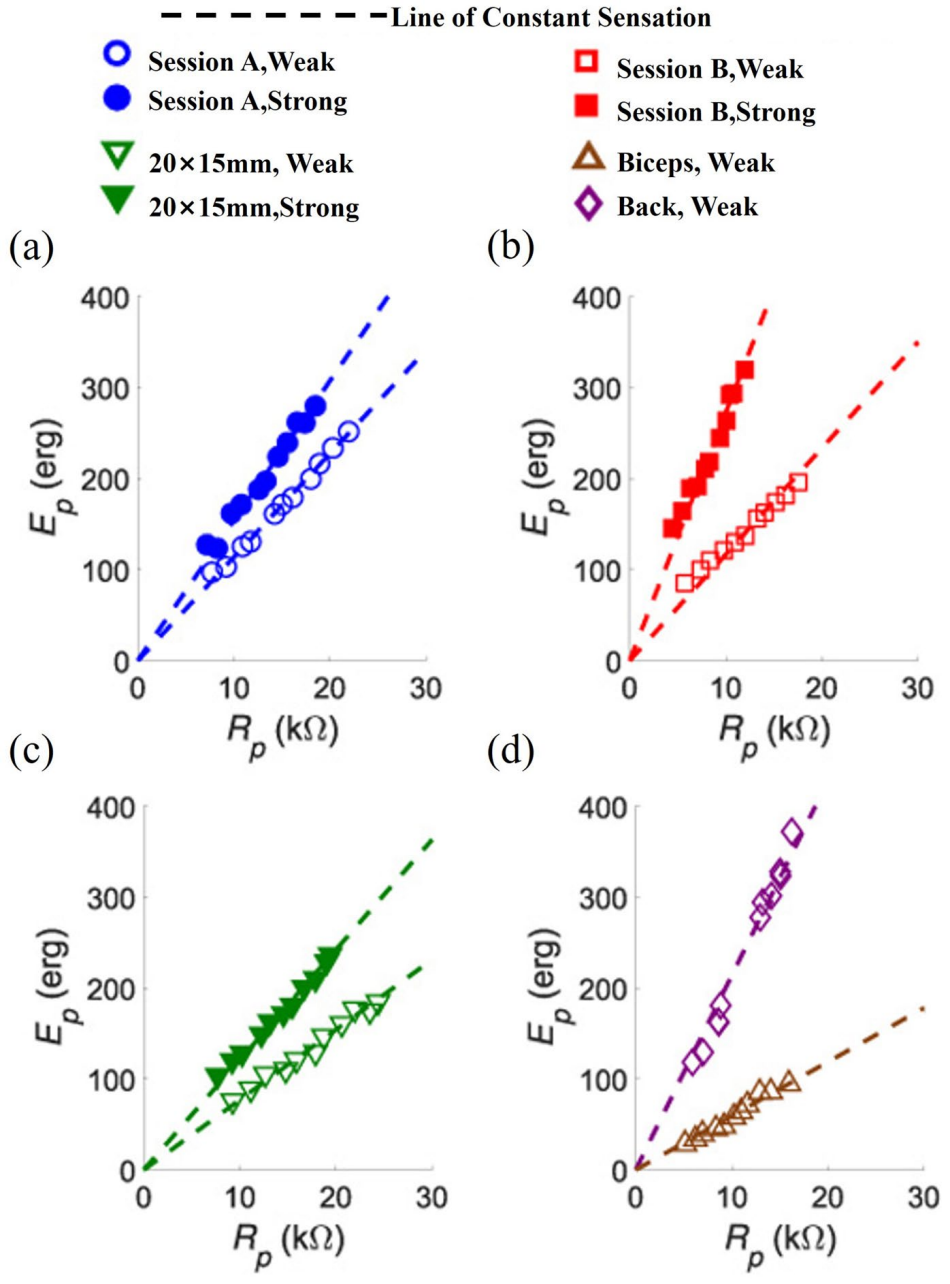
**(a–d)** Results from modelling the relationship of peak resistance (*R*_p_) to peak pulse energy (*E*_p_) and phase charge (*Q*) at constant sensation intensity (Akhtar et al., 2018). Reproduced with permission.

Although vibrotactile sensing has been used earlier in prosthetic control, electrotactile sensing has gradually received more attention as the need for high-resolution, low-latency haptic feedback has increased. As shown in Figures 12a–c, commercial vibrotactile-based devices, such as the H-glove, Dexmo glove, and HaptX glove, tend to have actuators that are bulky, limiting the spatial resolution for integration into portable or wearable devices (Varga, 2018; Perret et al., 2017). Electrotactile stimulators, due to their small size and flexibility, are able to provide multi-channel feedback and support closed-loop control of complex devices with higher resolution and response speed (Wang et al., 2021; Strbac et al., 2016). These advantages make electrotactile ideal for virtual tactile rendering, especially in portable and wearable device applications. To further optimize the performance of electrotactile sensing, Kajimoto (2012) proposed a real-time impedance feedback with pulse-width modulation technique for reducing the variation of tactile sensory intensity during stimulation. Despite the success of this technique in coping with impedance changes, the consistency of the model was still insufficient. Akhtar et al. (2018) improved the model and experimentally verified that the new method could better cope with the problem of impedance changes induced by poor electrode contact and the correlation coefficient was significantly improved (*r*^2^ > 0.9), as shown in Figures 13a–d.

**Figure 14:**
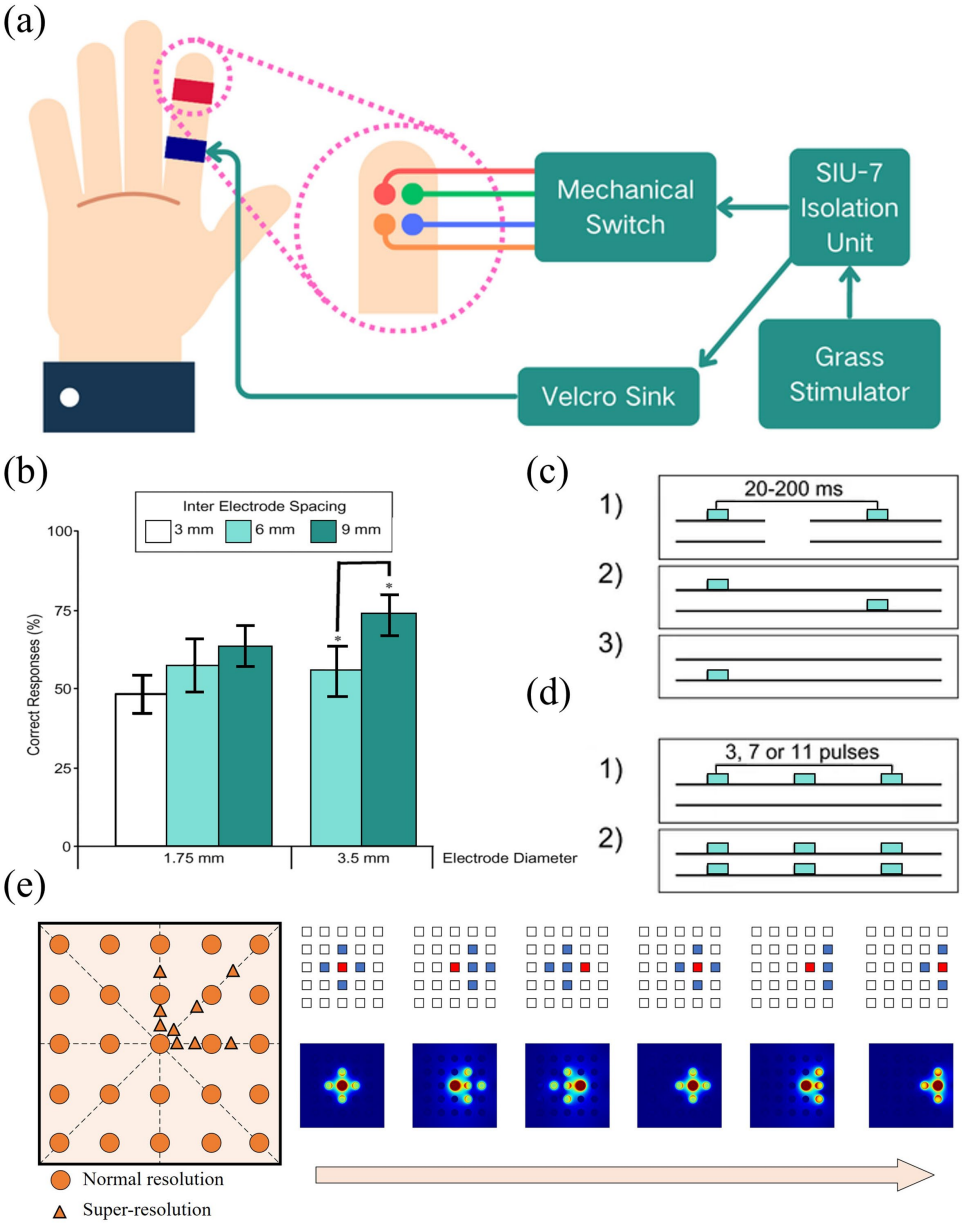
**(a)** Diagram of stimulator connections. **(b)** Chart of percentage correct responses for electrode size and interelectrode spacing (Warren et al., 2008). **(c)** Stimulation conditions in the effect of stimulus spacing on two-point discrimination. **(d)** Stimulation conditions in the effect of stimulus sequence length on two-point discrimination (Boldt et al., 2014). **(e)** Illustration of the 5 × 5 electrotactile device rendering resolution (left) and the top view of simulation results of current density under the different distribution of stimulation electrodes (right) (Lin et al., 2022). Reproduced with permission.

In virtual tactile rendering, spatial resolution is a crucial factor. Warren et al. (2008) showed that electrode spacing is the main factor affecting spatial resolution, while electrode area and stimulation frequency have relatively small effects, as shown in Figures 14a,b. Boldt et al. (2014) investigated the two-point recognition threshold of virtual tactiles and found that it is related to the pulse width and amplitude of the microcurrent, and the two-point recognition threshold can be effectively improved by controlling these parameters, as shown in Figures 14c,d. By optimizing the design of the electrode array, Lin et al. (2022) developed a high-resolution electrotactile system that was able to increase the total number of stimulation points from 25 to 105, which significantly improved the spatial resolution (76 points/cm^2^) and refresh rate (4 kHz), as shown in Figure 14e. The system is not only able to cover the entire range of human tactile intensities, but also solves the safety concerns of previous electrotactile reliance on high-voltage pulses. Although electrotactile devices show many advantages in virtual tactile rendering, they still face some limitations. Electrotactile sensation is difficult to stimulate slow-adaptive (SA) mechanoreceptors alone, leading to limitations in simulating sustained pressure perception. In addition, further miniaturisation of electrotactile devices and their integration with other tactile feedback technologies in the future will open up new possibilities for providing more realistic tactile sensations. The combination of these technologies is expected to enhance the accuracy and immersion of virtual tactile sensation, thereby providing a richer tactile feedback experience.

#### 3.2.4 Tactile interaction devices

Over the past 30 years, computing platforms have evolved through three major phases: the personal computer era, the mobile Internet era and the virtual reality (VR) era based on wearable computing devices. Tactile interaction devices, as a technology closely related to computing platforms, have also gone through three evolutionary phases: desktop tactile, surface tactile, and wearable tactile (Dangxiao et al., 2019). In the desktop tactile stage, the interaction mainly relies on multi-joint force feedback devices. The user interacts with the virtual environment by operating a robotic arm, and the force feedback is transmitted to the hand so that the user feels the virtual tactile sensation of the object. Typical representatives of such devices include Force Dimension and SensAble.

With the development of tactile technology entering the surface tactile stage, electrotactile interaction is beginning to receive more attention. Surface tactile aims to simulate the direct contact between the finger and the object, and the user can feel the texture and shape of the virtual object by sliding the finger on the touch screen and performing gesture operations such as zooming, panning, and rotating. Most current surface tactile devices use rigid printed circuit boards (PCBs) to build electrode arrays, with research focusing on spatial recognition, materials and surface texture roughness. For example, Liu et al. (2010) from Shanghai Jiaotong University designed a system that generates tactile feedback through electrical stimulation for delivering Braille information. The user can perceive Braille by touching the electrode array (Xin-yu, 2009). In addition, Germani et al. (2013) developed a novel tactile display capable of obtaining material roughness and texture feedback by touching the panel. However, surface tactile devices require the user to concentrate and actively apply some pressure to make contact with the display. This interaction is limited to a two-dimensional plane, making it difficult to meet the demands of complex hand movements in free space. With the rise of VR technology, surface tactile is no longer able to fully support multi-degree-of-freedom hand tracking.

**Figure 15:**
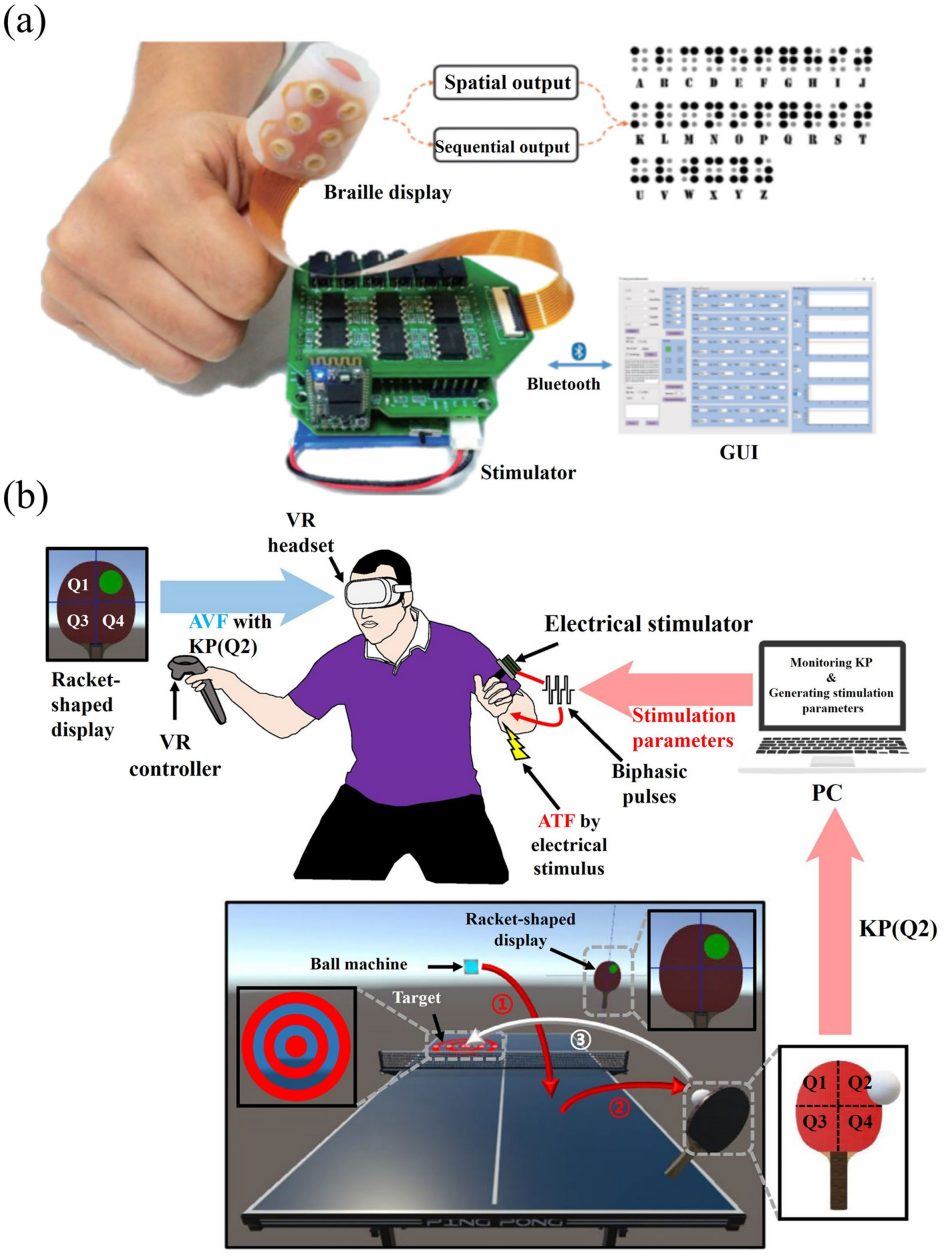
**(a)** The framework of Braille reading system (Zhou et al., 2022). **(b)** Overall illustration of the system implementation (Oh et al., 2024). Reproduced with permission.

Nowadays, wearable tactile devices have become the main research direction of tactile interaction devices. By wearing a tactile glove, the user can achieve a variety of gestures such as grasping, pinching, etc. to control the virtual hand-shaped avatar. Zhou et al. (2022) developed a portable Braille reading system based on electrotactile display technology, with electrodes made of flexible printed circuits (FPCs), which are lightweight and pliable, and can be worn by the user on his right thumb for Braille reading, as shown in Figure 15a. In addition, Pamungkas and Ward (2013) developed an electrotactile glove applied to the dorsal region of the hand for teleoperation and virtual haptic feedback, and Oh et al. (2024) designed a virtual ping-pong game combined with a VR device to enhance the user’s motor performance through electrotactile feedback as shown in Figure 15b. These wearable electrotactile interaction devices show great potential in areas such as education, rehabilitation therapy, and commercial activities. Importantly, by addressing critical issues such as electrode stability, stimulation uniformity, and adaptive feedback modulation, these technologies not only enhance user comfort and realism but also significantly expand the practical applicability of electrotactile systems, paving the way for broader commercialization and adoption. With the advancement of technology, wearable tactile devices are expected to play a more important role in more application scenarios in the future.

### 3.3 Application of virtual tactile presentation technology

Stimulation of the skin by an electric current excites the mechanoreceptors to generate action potentials, a virtual tactile feedback technique used to simulate tactile perception (Barfield and Furness, 1995). According to the definition of sensory information source by J. Loomis and S. Lederman, tactile sensation is the action of mechanoreceptors on afferent nerves to convert different forms of external stimuli into transmembrane potentials (Welch and Warren, 1986). The virtual tactile technology explored in this paper is mainly generated through electrical stimulation simulation and is widely used in medical rehabilitation, immersive entertainment, interactive experience, training and industrial design. Virtual tactile sensation can not only provide users with realistic sensory experiences, but also replace actual contact to a certain extent, thus bringing more convenience and innovation to life and work.

#### 3.3.1 Medical treatment and rehabilitation

##### 3.3.1.1 Surgical simulation

**Figure 16:**
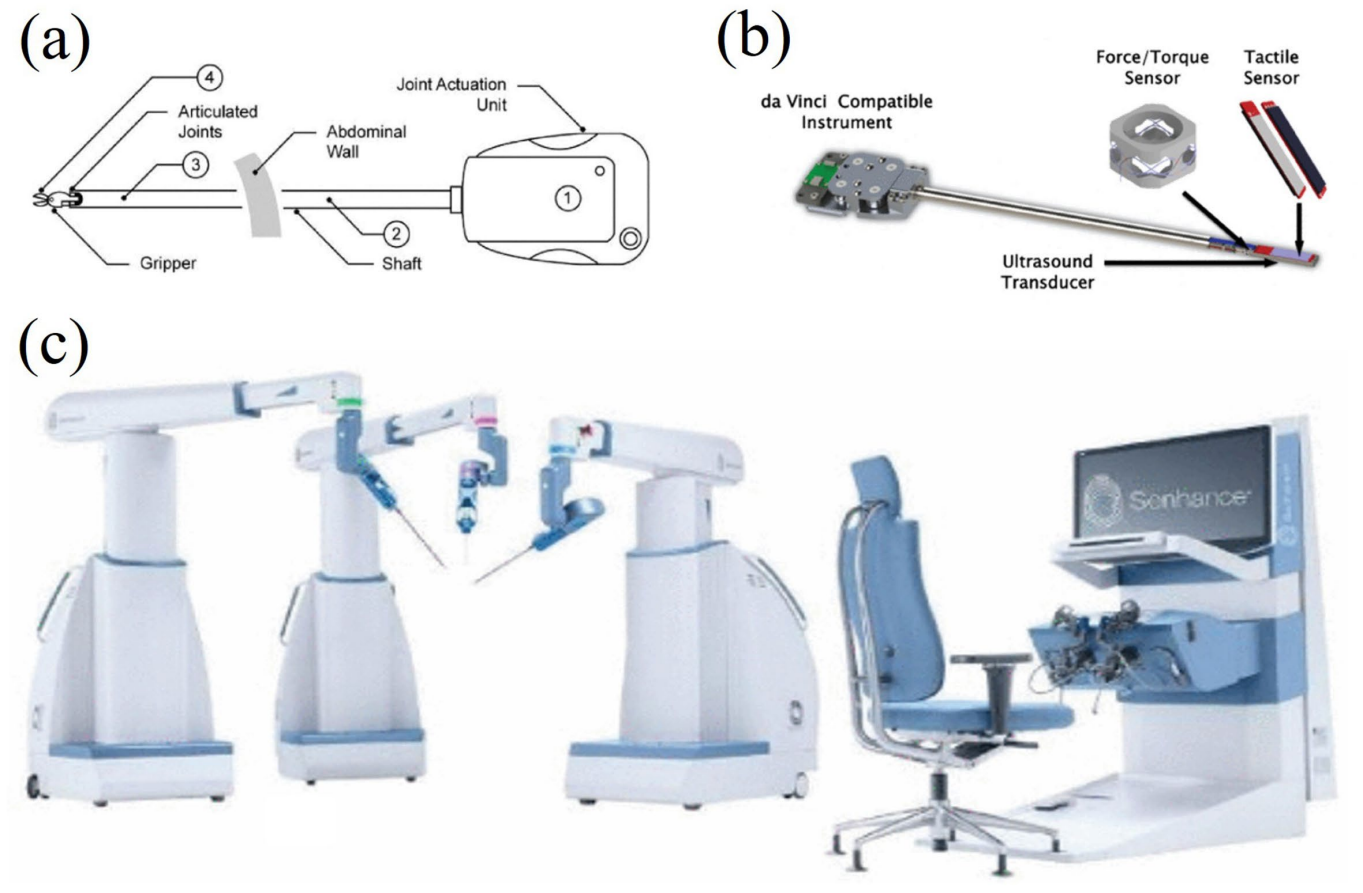
**(a)** Force sensor positions for the surgical hand and material options. **(b)** Bimodal palpation instruments for the da Vinci Classic Surgical System. **(c)** Surgeon’s console for the da Vinci Xi system (Patel et al., 2022). Reproduced with permission.

Virtual tactile technology plays an important role in surgical training and can provide surgeons with a safe and reproducible training environment through precise tactile feedback, helping them to improve their operational skills and reduce risks during surgery (Patel et al., 2022; Al-Ayyad et al., 2023). Traditional surgical training relies on clinical practice and animal models with ethical issues and operational risks. Virtual tactile devices provide a safe way to train without patients or animal models, greatly improving the feasibility and safety of training (Patel et al., 2022; Andaluz et al., 2016). For example, Patel et al. (2022) showed that a telesurgical system incorporating virtual tactile feedback significantly improved the safety and accuracy of surgery, as shown in Figure 16. Among the available systems, the da Vinci Surgical System is the most successful commercially available surgical robotic system. The latest model, shown in Figure 16c, has integrated tactile feedback to improve operating accuracy. As shown in Figures 16a,b, a variety of tactile feedback modalities are demonstrated, including skin stretching and vibration feedback. Among them, microcurrent-based tactile feedback can accurately sense surface texture and roughness, helping surgeons to simulate complex surgeries more accurately in virtual environments, improving their operating skills and success rates. However, current electrotactile systems face limitations in replicating the nuanced force gradients encountered in real tissues. Traditional methods, such as cadaveric training, provide direct haptic feedback with high fidelity but are constrained by ethical concerns and limited availability. Additionally, the high cost of advanced systems like the da Vinci Surgical System (Figure 16c) limits accessibility, particularly in low-resource settings. Further miniaturization and cost reduction are critical for widespread adoption.

**Figure 17:**
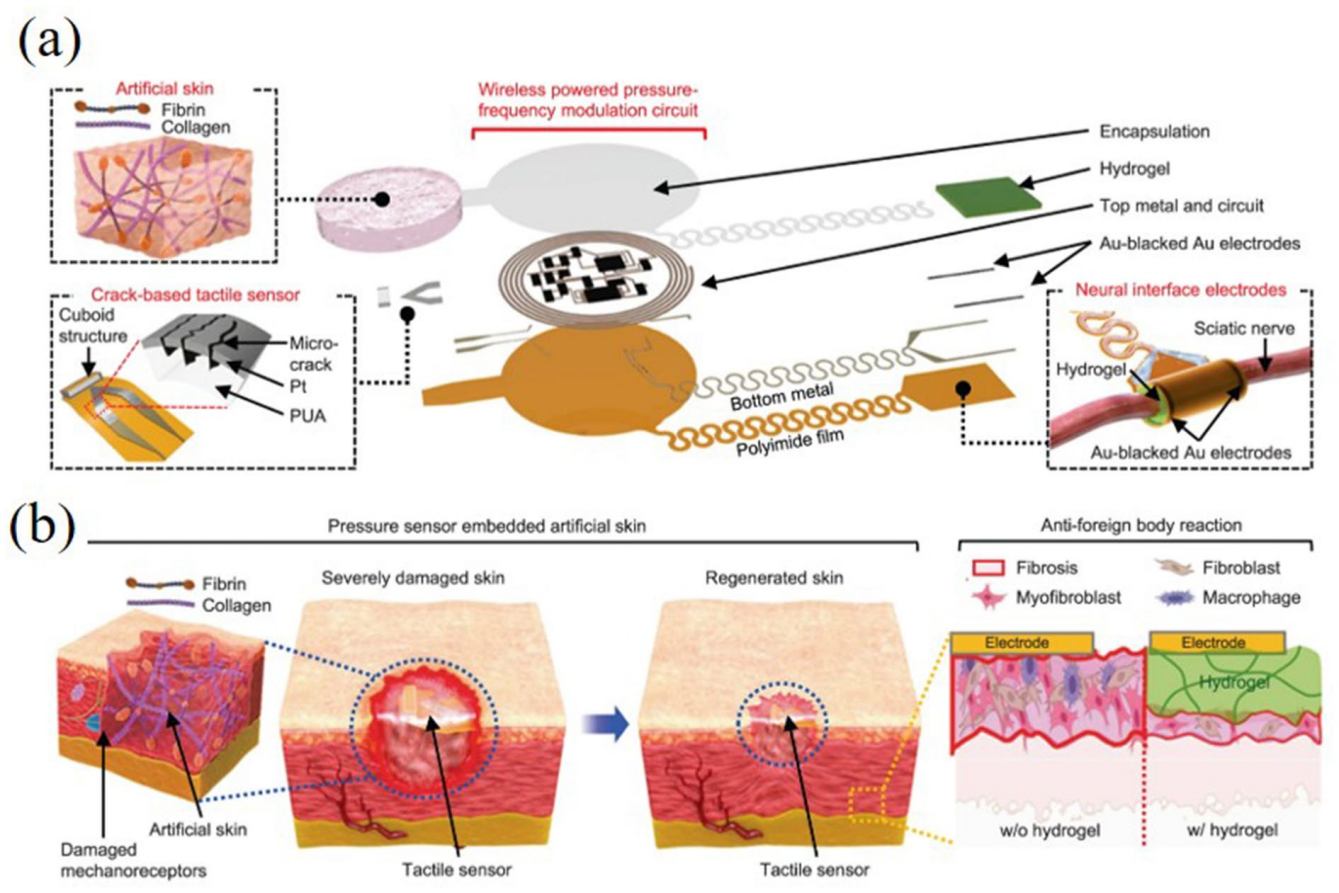
Overall schematic and converted tactile signal transfer process to stimulate the sciatic nerve (Dibben et al., 2023). **(a)** An exploded schematic illustration of the WTSA, consisting of artificial skin, a crack-based tactile sensor, a WPPFM circuit, neural interface electrodes, and a fibrin coating designed to reduce foreign body reactions. **(b)** A rapid skin regeneration process facilitated by an ECM-inspired artificial skin made of collagen and fibrin. Reproduced with permission.

In addition, the development and application of virtual tactile technologies have provided new tools and ideas for surgical treatment and postoperative rehabilitation, demonstrating significant results in restoring tactile function, accelerating wound healing, and improving the overall patient experience during surgery and rehabilitation. For example, Kang et al. (2024) introduced a fully implantable wireless tactile sensing system applied to bionic artificial skin specifically designed to promote wound healing and restore tactile function to the skin. The study showed that the system has the ability to work stably *in vivo* for long periods of time, opening up new possibilities for clinical treatments. The WTSA (shown in Figures 17a,b) consists of collagen- and fibronectin-based artificial skin (CFAS) that functions in conjunction with wirelessly-powered pressure-frequency modulation (WPPFM) circuits, neural-interface electrodes, and multilayer encapsulation technology. Through wireless power transfer, the system converts tactile signals (resistance changes) into sawtooth wave pulse signals at different frequencies to stimulate the nerve. The study specifically highlights the potential of the WTSA system in rehabilitation training, providing more efficient and accurate tactile recovery compared to traditional methods. The manufactured WTSA not only replaces severely impaired tactile function with biocompatible materials, but also promotes skin wound healing and regeneration using collagen and fibronectin-based artificial skin (CFAS). In addition, the hydrogel coating on the neural interface electrodes helps to minimise foreign body reactions, further enhancing the system’s clinical application prospects. In a broader application, the integration of wireless tactile systems with virtual reality environments can also be used to simulate a variety of realistic scenarios and complex operations, enabling patients with impaired tactile senses to be better equipped to cope with the real world (Dibben et al., 2023).

The application of virtual tactile technology in surgical training has achieved remarkable results. Tactile feedback systems based on microcurrent stimulation not only simulate realistic tactile sensations during surgery, but also provide higher response efficiency in terms of surface texture and roughness. This system allows surgeons to judge material properties and perform realistic surgical exercises more accurately in a virtual environment (Wang et al., 2017). This technique not only improves surgeons’ operating skills, but also helps them to quickly familiarise themselves with new techniques and equipment (Okamura, 2009). With the help of virtual tactile sensation, surgeons can practice various surgical operations, which dramatically improves the success rate and safety of surgery (Cao and Cerfolio, 2019).

##### 3.3.1.2 Rehabilitation therapy

**Figure 18:**
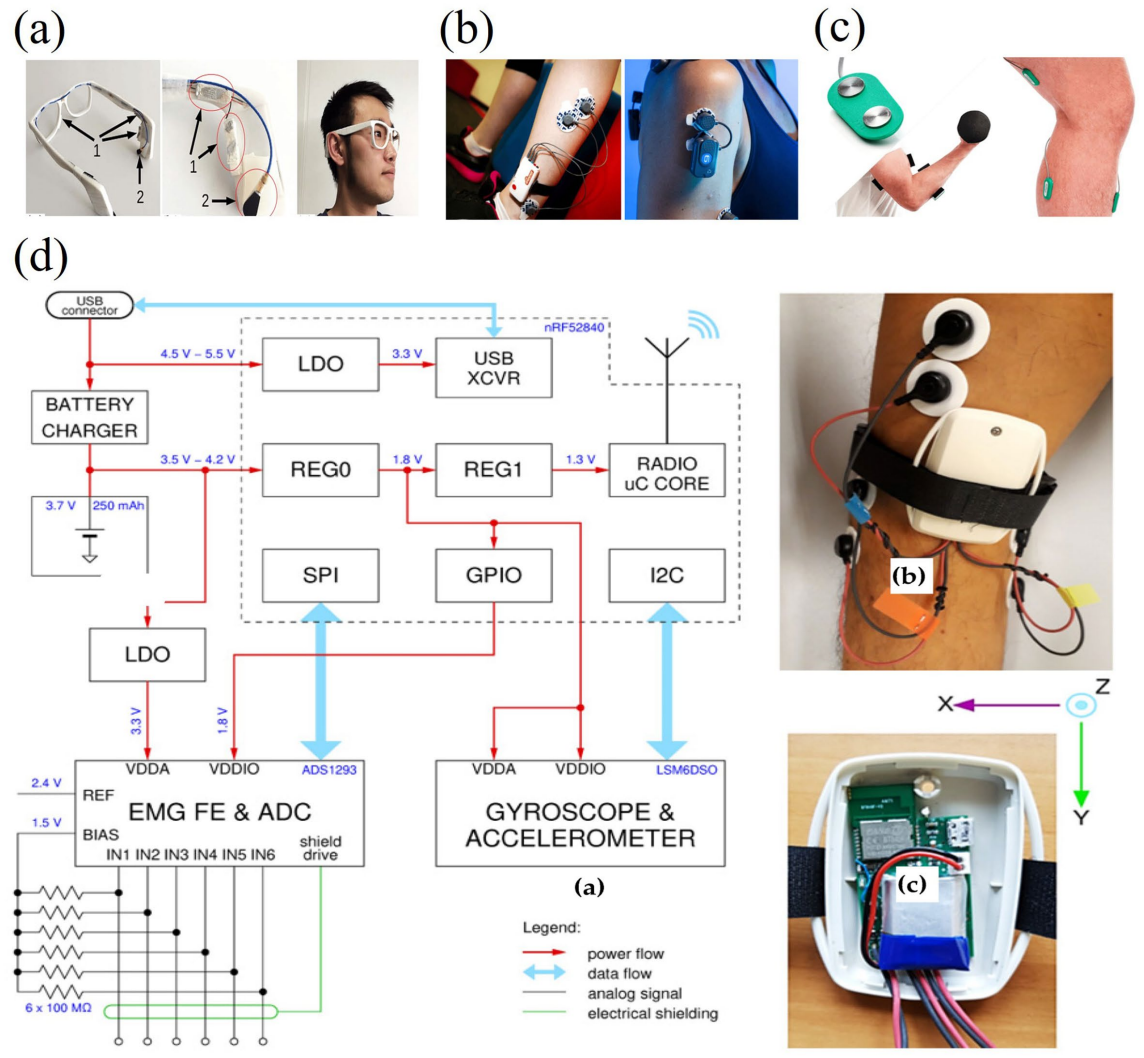
Model and application practice of virtual tactiles in rehabilitation therapy (Al-Ayyad et al., 2023). **(a)** Prototype of 3D printed smart glasses, presented in figure, Bone vibration sensors and muscle electrodes are integrated on the right temple. **(b)** Shimmer3 EMG and FreeEMG sensors in the leg and shoulder for monitoring body movements. **(c)** Biometrics Ltd EMG sensor and system (Muhsin et al., 2023). **(d)** Sensor node architecture with different power subsystems and circuit models. Reproduced with permission.

Virtual tactile technology has been widely used in rehabilitation therapy, especially in the rehabilitation of stroke patients and hand rehabilitation (Amirabdollahian et al., 2017; Broeren et al., 2004). Electrotactile feedback devices can help patients with hand and upper limb rehabilitation and promote nerve and muscle recovery (Figures 18a–c). For example, a clinical trial by Demeco et al. (2023) demonstrated that stroke patients using a system with electrotactile feedback significantly outperformed patients receiving conventional therapy in terms of motor skill recovery. Hand rehabilitation is an important application area of electrotactile technology (Hussain et al., 2020), and by providing interactive tactile feedback, patients can perform a variety of motor exercises in a virtual environment, thereby facilitating recovery. A robotic system incorporating virtual reality technology developed by Riener et al. (2005) significantly improves the rehabilitation by providing realistic tactile and visual experience outcomes and patient engagement. Compared to conventional physical therapy, electrotactile feedback enables personalized and adaptive training through real-time adjustments. However, prolonged use of wearable electrodes often causes skin irritation due to prolonged current exposure, and inconsistent contact impedance leads to unstable feedback during dynamic movements. Traditional rehabilitation tools, such as mechanical resistance devices, offer robust durability but lack the flexibility to simulate complex virtual scenarios. Moreover, the integration of multimodal feedback (e.g., combining electrotactile and vibrotactile) remains computationally intensive, resulting in latency (>50 ms) that disrupts the closed-loop sensory-motor cycle. Addressing these challenges requires advancements in biocompatible materials and low-latency signal processing architectures.

In the field of cardiac rehabilitation, virtual tactile technologies show great potential, especially high-intensity interval training (HIIT) combined with electrotactile feedback. This type of training significantly improves the cardiovascular health and functional capacity of cardiac patients. It also increases their motivation to recover. Real-time tactile feedback allows patients to more precisely control the intensity and pace of their training, thus optimizing rehabilitation outcomes. In addition, virtual tactile technology has shown value in developing assistive tools for visually impaired patients, improving their independence and quality of life through real-time electrotactile feedback. Notably, virtual tactile technology also has potential in the field of psychosocial rehabilitation, where virtual reality combined with tactile feedback can create immersive therapeutic environments that can help to alleviate patients’ pain and anxiety (as shown in Figure 18) (Mohan et al., 2021). While virtual tactile systems enhance engagement in cardiac rehabilitation, their effectiveness is highly dependent on user compliance and adaptability. Traditional supervised exercise programs ensure direct clinician oversight but lack the scalability of virtual solutions. A critical limitation lies in the oversimplification of tactile cues for cardiovascular feedback; current systems primarily modulate intensity rather than mimicking physiological nuances like arterial pulsatility. For psychosocial applications, although immersive VR environments reduce anxiety, the absence of thermal and proprioceptive feedback in electrotactile systems diminishes the realism of therapeutic scenarios. Future systems needintegrate multisensory modalities to bridge this gap.

#### 3.3.2 Immersive entertainment and interactive experience

Virtual tactile plays an important role in immersive entertainment and interactive applications such as virtual reality (VR) and augmented reality (AR). With the development from mechanical tactile devices to electrotactile and neural interface technologies, the applications of virtual tactile sensation have been expanding, gradually changing the way users interact with virtual environments (Raspopovic et al., 2014; Akhtar et al., 2018; Oh et al., 2024; Mohan et al., 2021). Compared with earlier devices that relied on mechanical moving parts to generate tactile sensations such as vibration, pressure, and friction, modern electrotactile technology has become a mainstream research direction by directly stimulating the skin’s nerve endings through microcurrents, which provides higher resolution and lower manufacturing costs. It is worth noting that compared with traditional mechanical tactile devices (such as systems that rely on vibration and electric stimulation to generate touch), although microcurrent-based virtual tactile technology shows obvious advantages in resolution and response speed, it still has significant technical limitations in terms of long-term stability, individual skin resistance differences, and insufficient stimulation of slow adaptive receptors. In addition, the existing systems often need to make a trade-off between hardware complexity and user portability in order to be compatible with complex hand movements and real-time feedback, which brings certain usage barriers and maintenance costs compared to traditional methods.

**Figure 19:**
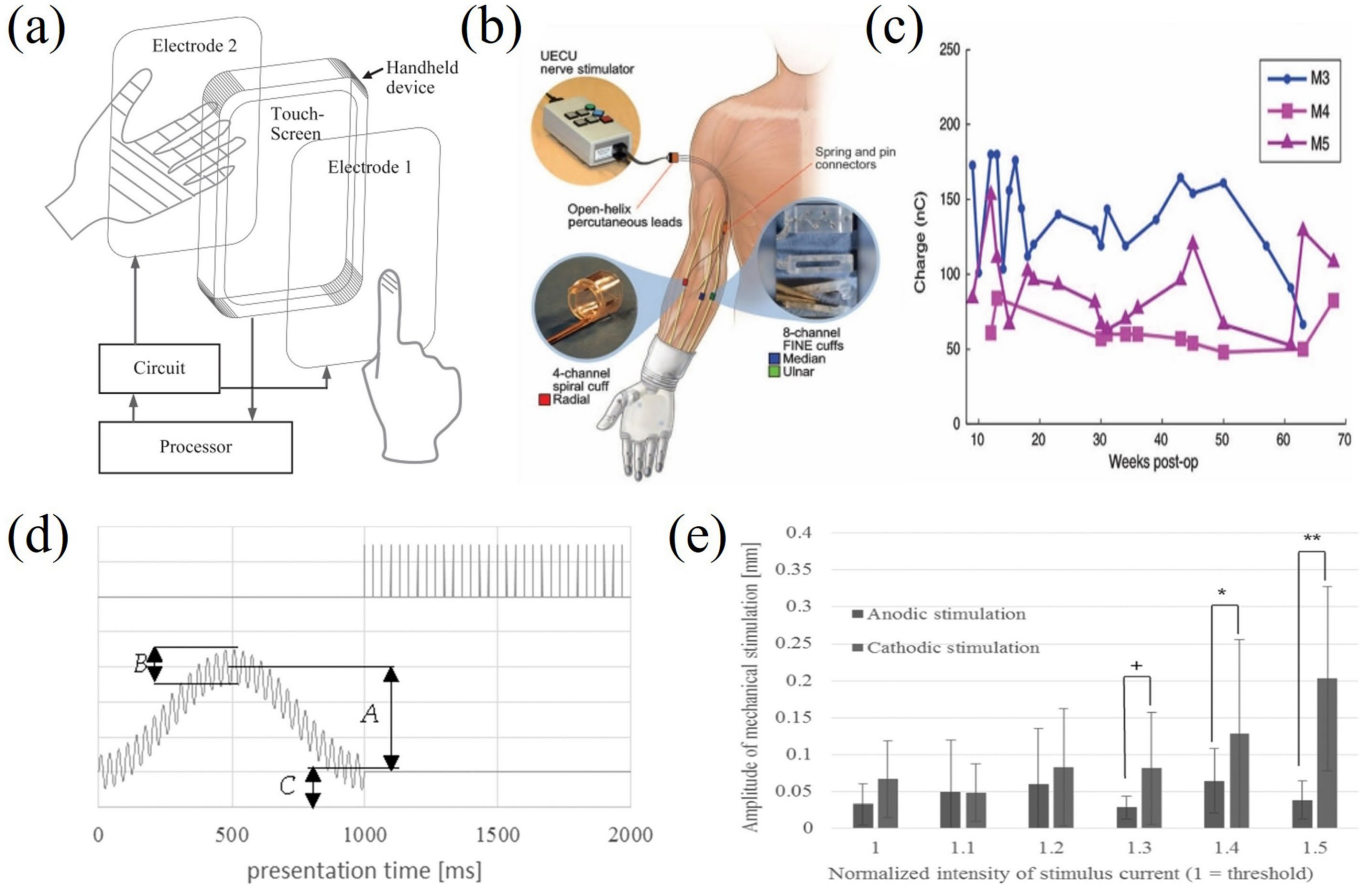
Research hotspots and applications of virtual tactiles in immersive entertainment virtual tactiles technology. **(a)** A schematic representation of the electrotactile display system with hand held device. The processor and the circuit unit are integrated into the hand held device (Pamungkas and Ward, 2016). **(b,c)** Schematic diagram of the basic structure of the implantable cuff and threshold tracking of the median channels M3, M4, and M5 up to 68 weeks showing the change in threshold over time (Tan et al., 2014). **(d)** Waveforms of the electrical stimulus pulses (top) and the mechanical stimulus vibration (bottom) used for the tactile sensation comparison experiment (Altinsoy and Merchel, 2011). **(e)** Relationship between the amplitude of mechanical stimulation and the intensities of anodic and cathodic stimulation (Altinsoy and Merchel, 2011). Reproduced with permission.

Currently, the research focus of virtual tactile technology is on different surfaces on a touchscreen device to enhance interactive immersion (as shown in Figure 19a) (Yem and Kajimoto, 2017). In addition, neural interface technology brings new possibilities for virtual tactile sensation. By directly stimulating nerves, the neural interface system developed by Tan et al. (2014) enables prosthetic users to have a near-natural tactile experience (as shown in Figures 19b,c). Similarly, Raspopovic et al. (2014) designed a bi-directional neural prosthetic system that provides tactile feedback in real time, enabling the user to feel the touch and pressure of objects.

Multimodal tactile feedback, on the other hand, further enhances the naturalness and subtlety of the user experience by combining electrotactile, mechanical, and vibrotactile sensations. For example, Yem and Kajimoto (2017) compared the tactile feedback effects of electrical and mechanical stimulation, providing a fundamental basis for future applications of haptic technology (as shown in Figures 19d,e) (Altinsoy and Merchel, 2011). Optimisation of haptic displays to enhance the tactile experience in virtual environments is also an important current research direction, with researchers working on the development of high-resolution, low-latency devices to provide more realistic tactile feedback (Dadi and Hariharan, 2018).

**Figure 20:**
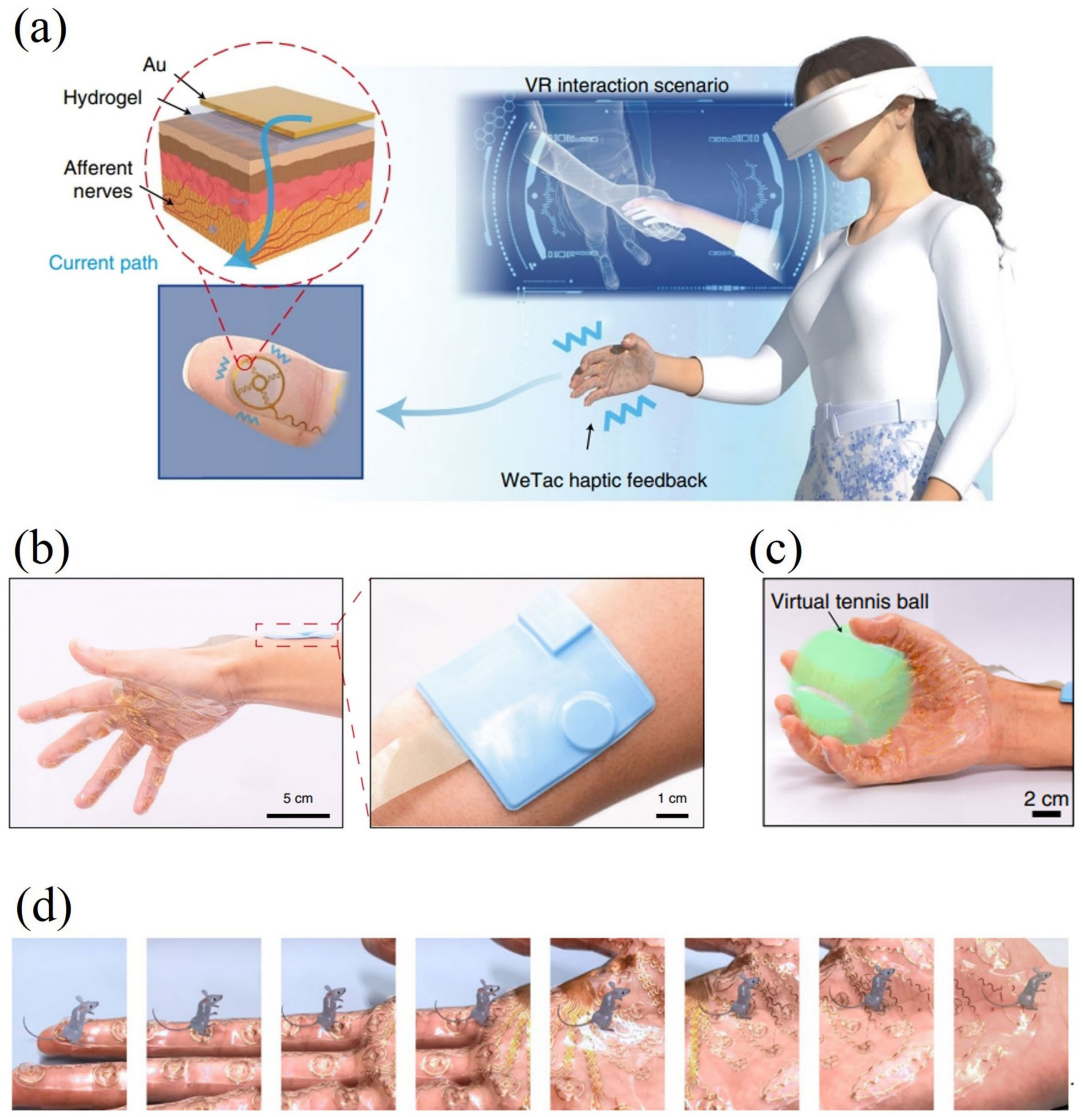
Research and practice on the application of virtual tactiles in immersive entertainment (Yao et al., 2022). **(a)** Schematic diagram of the mechanism of electrotactile application in immersive entertainment systems. **(b)** Photographs of the WeTac system with sensors worn on the hand with photographs showing the virtual experience hardware attached to the forearms. **(c)** AR scene showing a virtual tennis ball being grasped through feedback from the WeTac device. **(d)** AR scene showing a virtual mouse jumping forward and staying in each position of the hand for a period of time. Reproduced with permission.

In the future, virtual tactile technology has a wide range of application prospects in the field of immersive entertainment and interaction, and virtual tactile technology can greatly enhance the user’s sense of immersion and interactive experience. Through precise tactile feedback, players can feel the texture and weight of virtual objects, thus obtaining a more realistic gaming experience. For example, Yao et al. (2022) investigated a position-based electrotactile feedback system, which is a soft, ultra-thin, miniaturised radio-tactile system (WeTac) (Figure 20a) that induces tactiles by delivering electrical currents through the hand and acts as a tactile interface for skin integration. Its mapping of thresholds for different electrical parameters allows for personalized threshold data to be used to reproduce virtual touch sensations in the hand with optimized stimulus intensities to avoid causing pain. By precise control of sensory levels, time and spatial perception, it can provide personalized and precise electrotactile feedback when the user interacts with virtual objects (Figure 20b). For example for instance in the customised AR game they reproduced the tactile information of slowly grasping a tennis ball, where the user can accurately feel the exact position of the hit (Figure 20c), and additionally a virtual mouse feedback, where the mouse is standing on the user’s hand and moving forward to eat every piece of cheese in front of it, which are proofs of the virtual electro-tactile technology in AR scenarios thus improving the game’s interactivity and immersion (Figure 20d). The haptic device framework shown in Figure 20c not only reflects the integration advantages of the new flexible electrodes, but also reveals the differences in design compared to traditional rigid tactile interfaces. For example, although the use of flexible materials can improve wearing comfort and fit, it is also easily affected by skin movement, resulting in unstable contact, which is rarely seen in traditional mechanical devices.

In summary, although virtual electrotactile technology has shown great potential in improving immersive entertainment and interactive experience, and its high precision and low latency, which are superior to traditional tactile methods, have also attracted much attention, it must be clearly recognized that hardware complexity, individual differences, the stability of long-term contact between skin and electrodes, and the corresponding cost and maintenance issues are all key technical bottlenecks that need to be solved urgently. These limitations need to be focused on in future technical optimization, material upgrades, and control algorithm improvements in order to achieve complementary advantages between this technology and traditional methods and promote wider practical applications.

**Figure 21:**
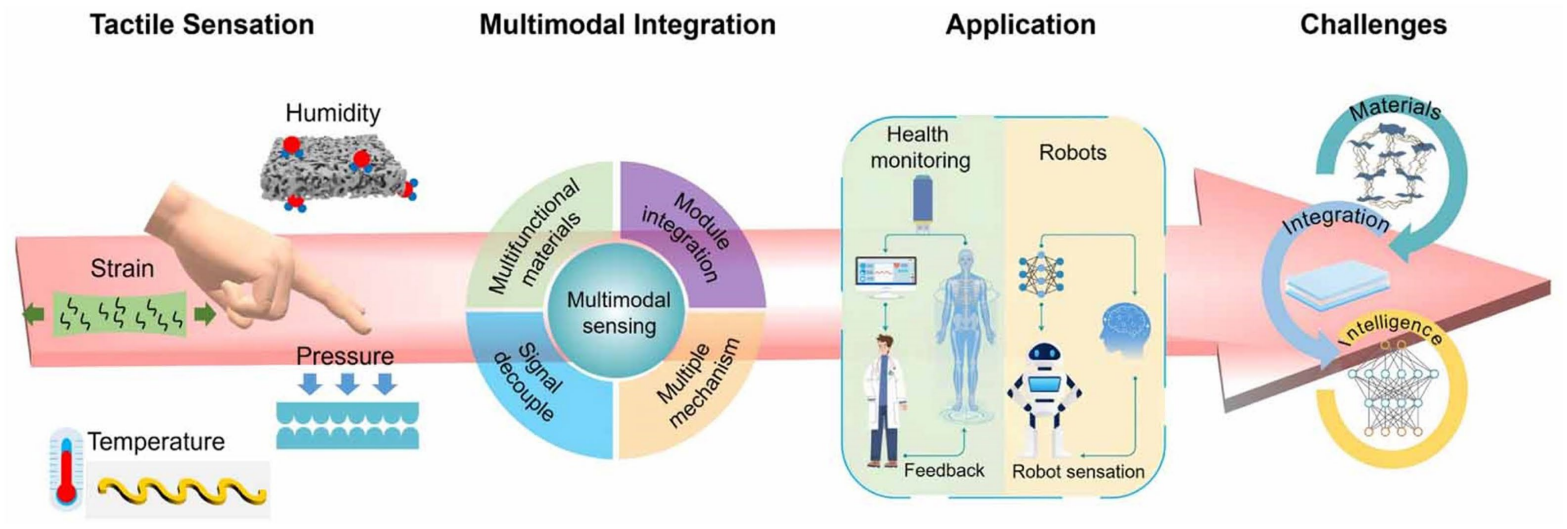
Tactile sensing mechanisms, multimodal integration, applications and challenges (Kong et al., 2024). Reproduced with permission.

In the field of immersive entertainment and interaction, virtual tactile technologies have undergone an evolution from mechanical tactile to electrotactile and neural interfaces, with a wide range of future application areas but also challenges. Electrotactile feedback has become a research hotspot for neural interfaces, multimodal tactile feedback and the optimisation of haptic display design for applications in virtual reality gaming, teleoperation and immersive VR interactions. Despite the strides made in this technology, its complexity and high cost remain barriers to diffusion. High-precision tactile devices require complex hardware and sophisticated manufacturing processes, which increase the cost and limit mass-market applications, e.g., although neural interface technology can provide highly realistic tactile feedback, its implantable devices are complex and costly (Pamungkas and Ward, 2016; Tan et al., 2014). The key to future developments will be to reduce device costs, improve accessibility of the technology, develop cost-effective hardware solutions, simplify production processes with new materials and manufacturing processes, and promote open-source hardware and software models (Figure 21).

#### 3.3.3 Education and training

**Figure 22:**
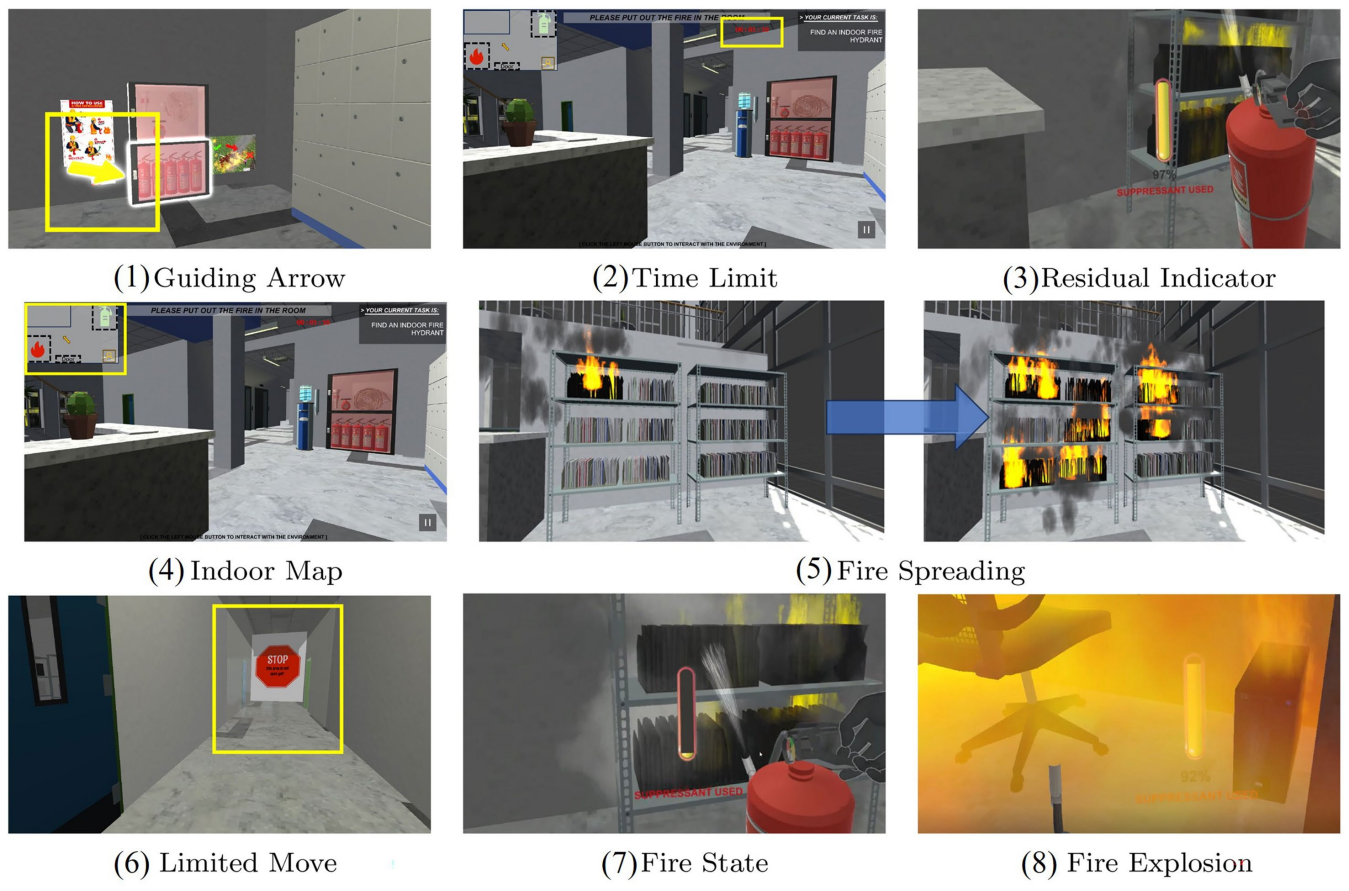
Virtual tactile-based simulation of fire escape process: (1)–(8) (Sun et al., 2024). Reproduced with permission.

The rapid development of virtual reality (VR) technology has opened up entirely new possibilities in the field of education and training, especially with the application of virtual tactile rendering technology, where learners are able to obtain immersive sensory experiences and real-time feedback in simulated virtual environments. This technology is not limited to traditional visual and auditory inputs, but also enhances learner engagement and learning through tactile feedback. For example, Medhat Alaker et al. investigated the application of virtual reality combined with tactile feedback in surgical training and found that by simulating real surgical scenarios, doctors can feel the physical feedback of surgical tools and tissues in real time, which leads to faster proficiency in surgical skills (Mao et al., 2021). Similarly, Sun et al. (2024) investigated the effect of virtual reality technology in emergency evacuation training, noting that simulating the fire escape process through tactile feedback can improve participants’ reaction speed and decision-making ability (as shown in Figure 22).

Virtual tactile technologies are also widely used in high-risk training scenarios. For example, in industrial assembly and maintenance training, by simulating complex assembly operations and providing tactile feedback, workers can practice and master key skills repeatedly in a safe virtual environment, which can effectively improve productivity and operational accuracy. In addition,

Taheri et al. (2017) explored the application of virtual reality in driver training and showed that by simulating realistic driving scenarios and providing tactile feedback, drivers can master complex driving skills faster, thereby improving driving safety and compliance with traffic rules. Traditional driving training often relies on physical driving simulators or actual road driving. These methods have advantages in realism and dynamic interaction, but are limited by environmental and economic costs. Virtual tactile technology provides a new means for driving training, which can adjust feedback parameters in real time. However, the current system has not yet reached the maturity level of traditional methods in terms of feedback accuracy and long-term stability, and urgently needs to be optimized to meet the strict standards of safety training.

The wide application of virtual tactile rendering technology in the field of training is not limited to the above areas. In medical training, virtual reality technology combined with tactile feedback is innovative in teaching anatomy. This allows students to improve their identification and manipulation skills by gaining a deeper understanding of human structure and organ function through simulated tactile experiences. In addition, Girardi et al. (2022) discussed the application of virtual reality in military training, where by simulating complex battlefield scenarios and providing tactile feedback, soldiers can practice tactical manoeuvres and weapon manipulation in real time, enhancing real-world capabilities and decision-making speed.

Although virtual tactile rendering technology provides students with an immersive and interactive learning experience in the field of education and training, and has obvious advantages in reducing safety risks and repeated training costs, it still faces many challenges in terms of system stability, tactile realism, personalized adaptability, and cost control compared to traditional training methods. Future research needs to make breakthroughs in hardware optimization, control algorithm improvement, and multimodal interactive fusion to better realize the complementary advantages of virtual technology and traditional teaching methods and promote the comprehensive innovation of education and training models. In the present day, the application of virtual tactile rendering technology in the field of training and training has demonstrated significant potential and effectiveness. By combining multiple inputs from vision, hearing and tactiles, this technology not only improves learners’ immersion and engagement, but also plays a key role in practical operations and skills training. With further advances in technology and the expansion of application scenarios, virtual tactile rendering technology is expected to become an important support tool in the field of training and coaching in the future, providing students, professionals and military personnel with more vivid, safe and efficient learning and training environments.

#### 3.3.4 Industry and design

##### 3.3.4.1 Product design and prototype testing

**Figure 23:**
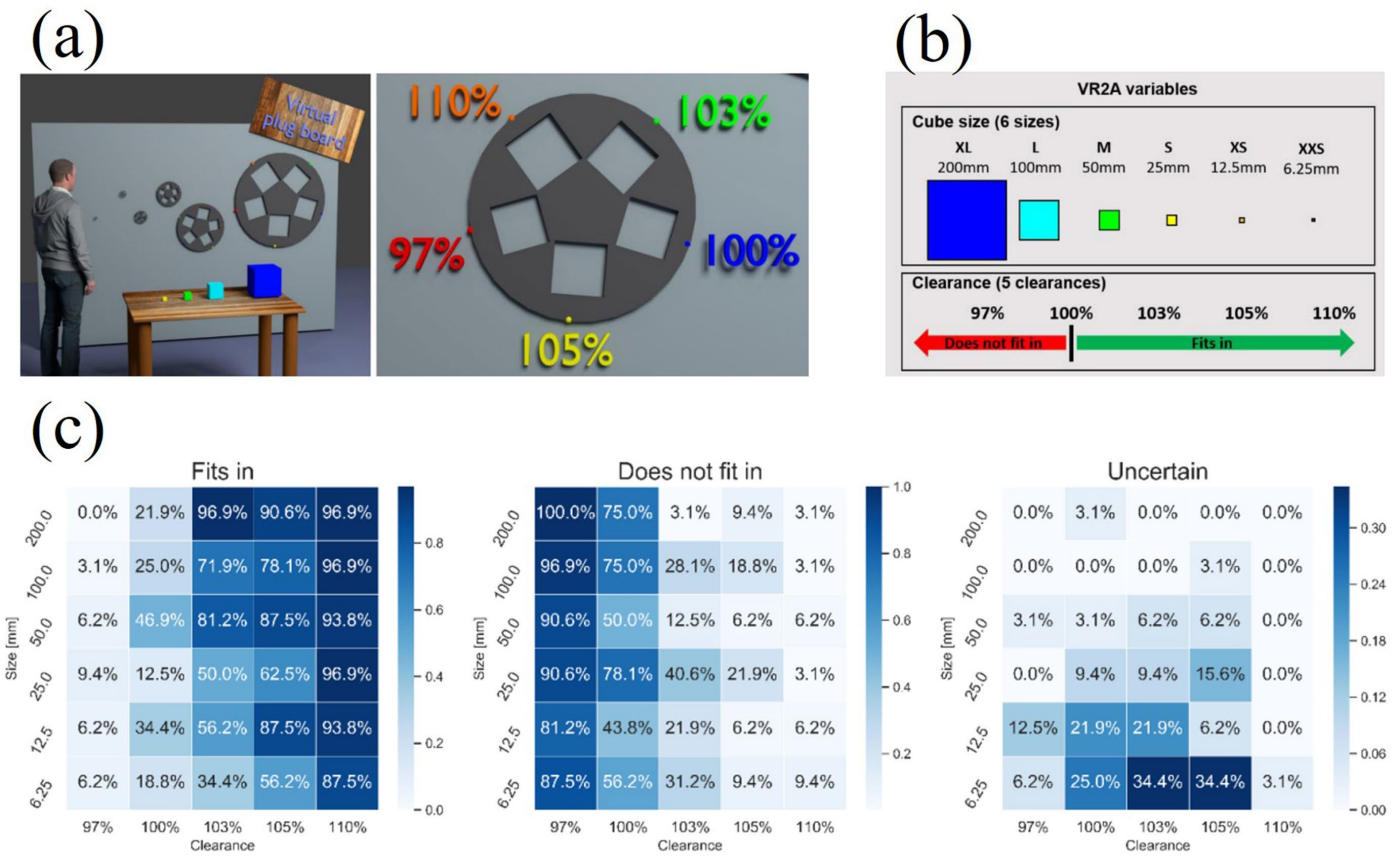
**(a)** Rendering of open virtual environments with six different sized cubes. **(b)** Overview of two standard experimental designs in VR environments with independent variables: size and gap. **(c)** Relative frequencies of participants in different scenarios of the VR2A benchmark test (Otto et al., 2019). Reproduced with permission.

Virtual tactile rendering technologies are increasingly used in product design and prototype testing. These technologies can improve the efficiency of designers and engineers’ decision-making and product quality during the design phase by simulating realistic tactile feedback. For example, it has been shown that by using a high-fidelity tactile feedback system, designers can more intuitively perceive and evaluate the physical characteristics of a product, such as the surface texture, hardness, and shape detail, as shown in Figure 23b (Girardi et al., 2022). This intuitive perception helps to reduce the cost and time of trial and error in design, thus speeding up the time to market. We can also note the relative frequency of participants in the VR2A benchmark test in different scenarios, and the enhancement of the experience and experimentation brought by virtual tactiles to the participants is evident with continuous training and adaptation (Figure 23).

In terms of prototype testing, virtual tactiles helps design teams to more accurately assess the actual experience of using a product by simulating the real sensations of users interacting with the product. For example, several studies have explored how tactile feedback systems can be utilised to assess the user-friendliness and operational comfort of control panels in car interiors (Breitschaft et al., 2019). By simulating the sensation of the user’s fingers on the control buttons, researchers were able to analyze the impact of different design options on user perception and operational efficiency, thereby optimizing the layout and functional design of the control panel. In addition, virtual tactiles can provide important feedback in the early stages of product design. By combining it with virtual reality technology, designers can explore and modify product concepts directly in a digital environment without relying on traditional physical prototyping (Dangxiao et al., 2019). This approach not only saves material and manufacturing costs, but also significantly shortens the product development cycle, allowing designers to be more flexible and responsive to changes in market demand. Compared with traditional physical prototype testing and manual design, virtual tactile rendering technology can shorten product design cycles and reduce material costs through real-time feedback. However, the current system still has obvious deficiencies in real touch reproduction, response delay, and multi-point tactile feedback consistency. For example, compared with traditional groping physical prototype testing, the fineness and dynamic changes of touch in the virtual environment have not yet met users’ expectations of the feel of real products, which to some extent affects designers’ intuitive judgment of product details.

##### 3.3.4.2 Remote operation and control

The application of virtual tactile rendering technologies is of great importance in the field of industrial automation and teleoperation. These technologies enable remote operators to sense and manipulate complex machines and equipment over a network while maintaining a high degree of control and feedback over the operating environment (Patel et al., 2022). For example, in the medical field, doctors can perceive and manipulate surgical robots in real time via virtual tactile systems to accurately perform minimally invasive surgical operations, while minimizing surgical risks and errors.

**Figure 24:**
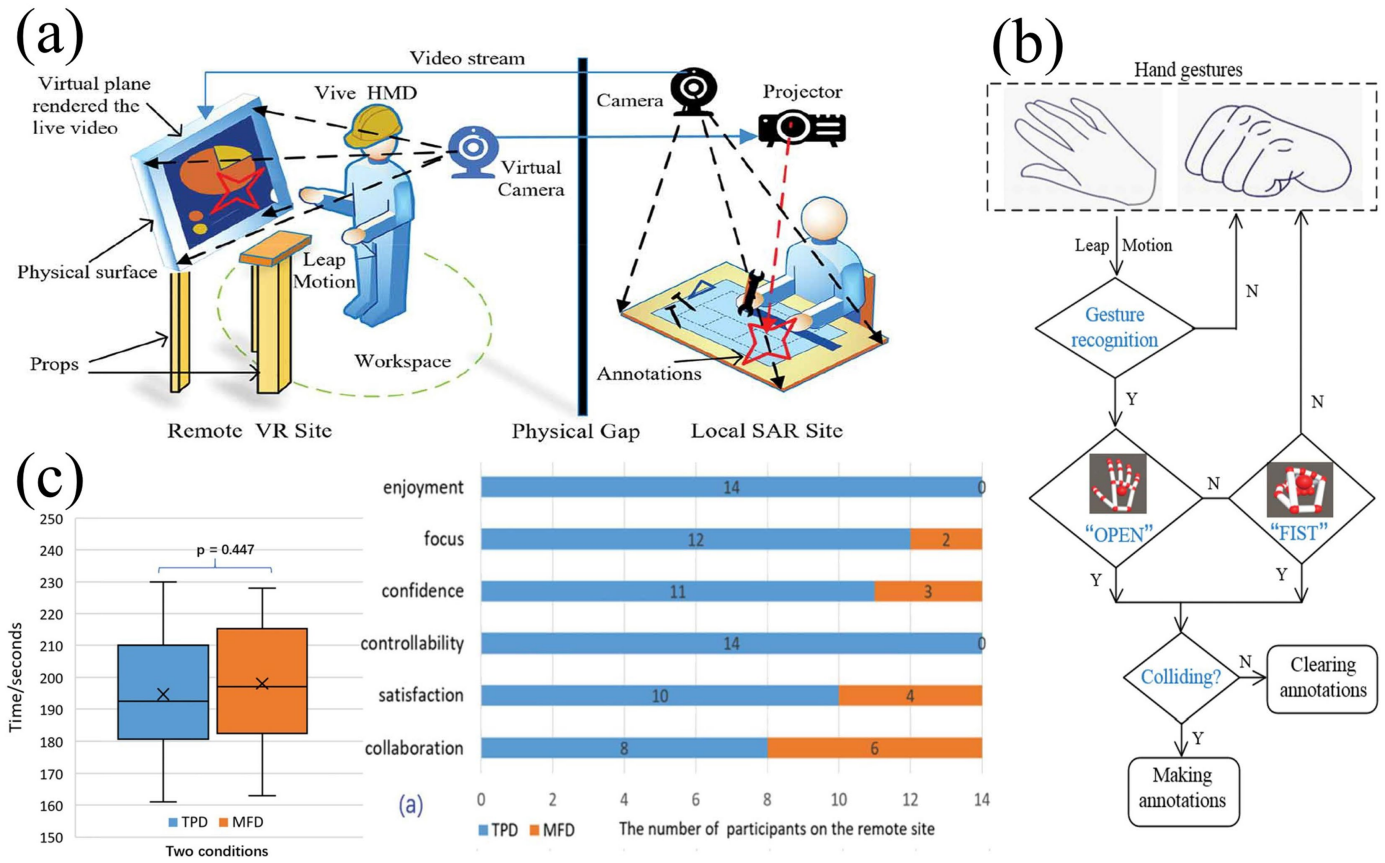
**(a)** Proof-of-concept diagram for TPD and MFD. **(b)** Shows the entire system architecture including hardware, software and overview of key data flows including SAR and VR sites. **(c)** Evaluating the performance of the difficulty level of the A/B model (Wang et al., 2020). Reproduced with permission.

Teleoperation is a technology that enables remote interaction between humans and machines, where the control end is local and the execution end is somewhere in remote space that cannot be directly perceived locally (Aiguo, 2013). In teleoperation systems, commands are given by a local human user to control the physical application of a remote machine ontology (Figure 24a). As opposed to intelligent programming, teleoperation systems construct realistic and reliable human-machine interaction scenarios that facilitate the control of robots to handle complex, urgent, or extreme tasks. In contrast, in traditional teleoperation techniques, fine manipulation control is challenging due to the lack of tactile feedback information, which prevents the local user from sensing the contact between the engineering robot and the ground or obstacles, which can easily lead to the tipping over of the real engineering robot. By realising realistic and delay-free tactile feedback, it is possible to realize more delicate robotic dexterity operations, such as pouring water, unscrewing bottle caps, opening cardboard boxes, spinning Rubik’s cubes, writing, helping people with headphones, and even human massage. In the field of human-computer interaction, Wang et al. (2020) proposed a virtual reality (VR)-spatial augmented reality (SAR) telecollaboration system, which provides tactile feedback through the tangible interaction between a local worker and a remote expert assistant, and Figure 24b also demonstrates the flow of his gesture transfer algorithm. The researcher conducted a within-subjects user study using this system to compare two remote collaborative interfaces between local workers and expert assistants, one for mid-air free-drawing (MFD) and the other for tangible-physical-drawing (TPD), as shown in Figure 24c. The results show no significant differences in terms of performance time and operational errors. However, users believe that a TPD interface supporting passive tactile feedback can significantly improve the user experience of remote experts in VR. From the perspective of traditional remote operation, which relies on direct manipulation of physical models to obtain real tactile feedback, although virtual tactile technology has achieved real-time interaction across regions, its feedback accuracy and response delay issues cannot be ignored. The current tactile feedback system is prone to errors under high-load conditions, and it is difficult to fully simulate the actual tactile experience caused by subtle changes in finger movements, which to a certain extent limits its practicality in industrial high-precision operation and equipment maintenance.

In general, virtual tactile presentation technology has shown advantages over traditional methods in terms of high efficiency, low cost and safety in the field of product design and industrial remote control, but it also faces technical bottlenecks such as insufficient hardware stability, poor tactile feedback authenticity and insufficient response to complex interactive environments. Future research needs to make breakthroughs in material innovation, feedback algorithm optimization and multimodal fusion to achieve complementary advantages between virtual tactile technology and traditional industrial practices and promote the widespread application of this emerging technology.

In addition, virtual tactiles technology can enhance engineers’ ability to operate during remote facility maintenance and troubleshooting. With real-time tactile feedback, engineers can remotely identify and address equipment faults and adjust operational strategies in a timely manner, thereby reducing maintenance time and costs (Rastogi and Kumar Srivastava, 2019). This capability is particularly important for companies that operate and maintain facilities across geographies and on a large scale, and can effectively improve overall operational efficiency and responsiveness. Nowadays, the application of virtual tactile rendering technology in the field of industry and design demonstrates a broad development prospect and important application value. As technology continues to advance and application scenarios expand, we can expect these technologies to continue to contribute to industrial production efficiency, product design innovation and remote operation safety in the future.

## 4 Trends for the future

In the future, the development of virtual tactile technology will be centered around several key directions to improve its accuracy, portability, and user experience, thereby facilitating broader applications across multiple fields. The novelty of this review lies particularly in its comprehensive synthesis of physiological and psychophysical mechanisms, advanced electrotactile rendering technologies, and emerging trends in device integration, providing an integrated theoretical and practical framework to guide future research and development efforts. Specifically, the establishment of more precise three-dimensional human tissue electrical stimulation conduction models will substantially enhance the accuracy of electrotactile feedback and multi-point stimulation, thereby expanding its utility in more complex tactile interaction scenarios.

Furthermore, addressing demanding application requirements, such as those found in virtual reality (VR) and augmented reality (AR) environments, necessitates the development of lightweight, flexible, and highly responsive electrotactile devices. Emphasis should be placed on miniaturizing microcurrent generators and selectors, optimizing electrode materials, and refining ergonomic designs to ensure comfort and long-term usability. Real-time impedance feedback combined with pulse-width modulation techniques will dynamically stabilize stimulation intensity, mitigating perceptual inconsistencies caused by impedance variability, and thus significantly enhancing the reliability and realism of tactile interactions.

Enhancing user experience further calls for robust advancements in multimodal integration techniques, especially in developing fusion control models and innovative audio-visual-tactile systems. Such integrated feedback mechanisms promise to yield richer, more immersive interaction scenarios. Practically, virtual tactile technologies are poised to see expanding applications within education and training, industrial design, medical rehabilitation, and related areas, highlighting the need for tailored solutions addressing sector-specific requirements.

Moreover, future studies could benefit from deeper exploration into the fundamental mechanisms underlying microcurrent-based tactile rendering, including the differences between physical and electrically induced tactile sensations, and the integration of multi-physical stimulation modes such as ultrasonic and mechanical interactions. Research into adaptive rendering techniques, leveraging individual physiological parameters to optimize tactile experiences, will be particularly valuable in creating personalized and contextually responsive feedback systems. Finally, the development and validation of advanced, wearable audio-visual-tactile platforms represent an essential frontier, encouraging interdisciplinary collaborations among researchers in neuroscience, material science, computational modelling, and human-computer interaction.

Advancing along these specific, targeted research trajectories will help virtual tactile technology mature further, ultimately enabling more natural, immersive, and effective human-computer interactions, with substantial implications for both theoretical research and practical innovation in diverse application domains.
